# Prostaglandin E_2_ glyceryl ester is an endogenous agonist of the nucleotide receptor P2Y_6_

**DOI:** 10.1038/s41598-017-02414-8

**Published:** 2017-05-24

**Authors:** Antje Brüser, Anne Zimmermann, Brenda C. Crews, Gregory Sliwoski, Jens Meiler, Gabriele M. König, Evi Kostenis, Vera Lede, Lawrence J. Marnett, Torsten Schöneberg

**Affiliations:** 1 Rudolf Schönheimer Institute of Biochemistry, Medical Faculty, University of Leipzig, 04103 Leipzig, Germany; 2 Department of Biochemistry, Chemistry and Pharmacology, Vanderbilt Institute of Chemical Biology, Vanderbilt-Ingram Cancer Center, Vanderbilt University School of Medicine, Nashville, TN 37232-0146 USA; 3 Department of Chemistry, Center for Structural Biology, Vanderbilt University, Nashville, TN 37232-8725 USA; 4 Institute of Pharmaceutical Biology, University of Bonn, 53115 Bonn, Germany; 5 Department of Biomedical Informatics, Vanderbilt University School of Medicine, Nashville, TN 37232-8725 USA

## Abstract

Cyclooxygenase-2 catalyses the biosynthesis of prostaglandins from arachidonic acid but also the biosynthesis of prostaglandin glycerol esters (PG-Gs) from 2-arachidonoylglycerol. Previous studies identified PG-Gs as signalling molecules involved in inflammation. Thus, the glyceryl ester of prostaglandin E_2_, PGE_2_-G, mobilizes Ca^2+^ and activates protein kinase C and ERK, suggesting the involvement of a G protein-coupled receptor (GPCR). To identify the endogenous receptor for PGE_2_-G, we performed a subtractive screening approach where mRNA from PGE_2_-G response-positive and -negative cell lines was subjected to transcriptome-wide RNA sequencing analysis. We found several GPCRs that are only expressed in the PGE_2_-G responder cell lines. Using a set of functional readouts in heterologous and endogenous expression systems, we identified the UDP receptor P2Y_6_ as the specific target of PGE_2_-G. We show that PGE_2_-G and UDP are both agonists at P2Y_6_, but they activate the receptor with extremely different EC_50_ values of ~1 pM and ~50 nM, respectively. The identification of the PGE_2_-G/P2Y_6_ pair uncovers the signalling mode of PG-Gs as previously under-appreciated products of cyclooxygenase-2.

## Introduction

Prostaglandins are potent bioactive lipid messengers derived from arachidonic acid^1^. Cyclooxygenases (COXs) catalyse the rate-limiting step of prostaglandin biosynthesis. Besides this well-studied enzymatic function of COX isoenzymes, COX-2 selectively oxygenates 2-arachidonoylglycerol (2-AG) to form prostaglandin glycerol esters (PG-Gs)^2^. The initially formed PG-G endoperoxides are further transformed to PGE_2_-G, PGD_2_-G, PGF_2α_-G, and PGI_2_-G^3^. Despite its rapid degradation^4^, PGE_2_-G is detectable following activation of different macrophage cell lines^5–8^ and is present in rat paw after treatment with carrageenan^9^. This implicates PG-Gs as potential mediators of pain and the innate immune response.

Very little is known about the biological function of PG-Gs. PGE_2_-G induces hyperalgesia^9^, improves excitatory glutamatergic synaptic transmission, and promotes neurotoxicity in rat hippocampal neurons^10^. Previous work suggests that PGE_2_-G activates a G protein-coupled receptor (GPCR) in the murine macrophage-like cell line RAW264.7 and the human lung adenocarcinoma cell line H1819^11, 12^. The fast Ca^2+^ response observed with both cell lines indicates specific signal transduction via a G_q_- and/or G_i_ protein-coupled receptor. Interestingly, these studies revealed an extremely low EC_50_ value in the range of 1 pM for PGE_2_-G. Physiologically, this seems reasonable because PGE_2_-G occurs in low amounts and is rapidly hydrolysed to PGE_2_^4^. Indeed, stimulation of macrophages with lipopolysaccharide and zymosan induces synthesis of PGE_2_-G in amounts sufficient to activate the unknown PGE_2_-G receptor^7^.

Identification of the PGE_2_-G receptor is of great interest as a first step toward characterizing the physiological function of PG-Gs and to pharmacologically manipulate this signalling system. Since previous attempts demonstrated that PGE_2_-G does not efficiently activate the known prostanoid receptors EP_1–4_, DP, FP, TP, or IP^9, 11, 13^, we extended our search by screening all currently known orphan GPCRs for PGE_2_-G activation. However, this classical approach to identify the endogenous receptor for PGE_2_-G was unsuccessful. Therefore, we sequenced the transcriptome of several PGE_2_-G responder and non-responder cell lines using Illumina RNA sequencing technology. In a subtractive approach, we identified several GPCRs, which are significantly expressed in the PGE_2_-G responder cell lines. Cloning and functional testing of these receptors were performed and revealed the UDP receptor P2Y_6_ as the GPCR for PGE_2_-G.

## Results

### Screening of orphan GPCRs

Because previous studies failed to show binding or activation of PGE_2_-G at the known prostanoid receptors^9, 11, 13^, we attempted to identify a receptor among GPCRs which were considered orphan at this time. In a Path-Hunter^®^ biosensor Orphan GPCR cell line panel (DiscoveRx, USA), 78 orphan GPCRs were tested for their ability to be activated by PGE_2_-G. None of the tested receptors demonstrated a positive response (Supplementary Table S1).

### RNA sequencing reveals differentially expressed G_q/_G_i_ protein-coupled receptors in PGE_2_-G-responding cell lines

**Figure 1:**
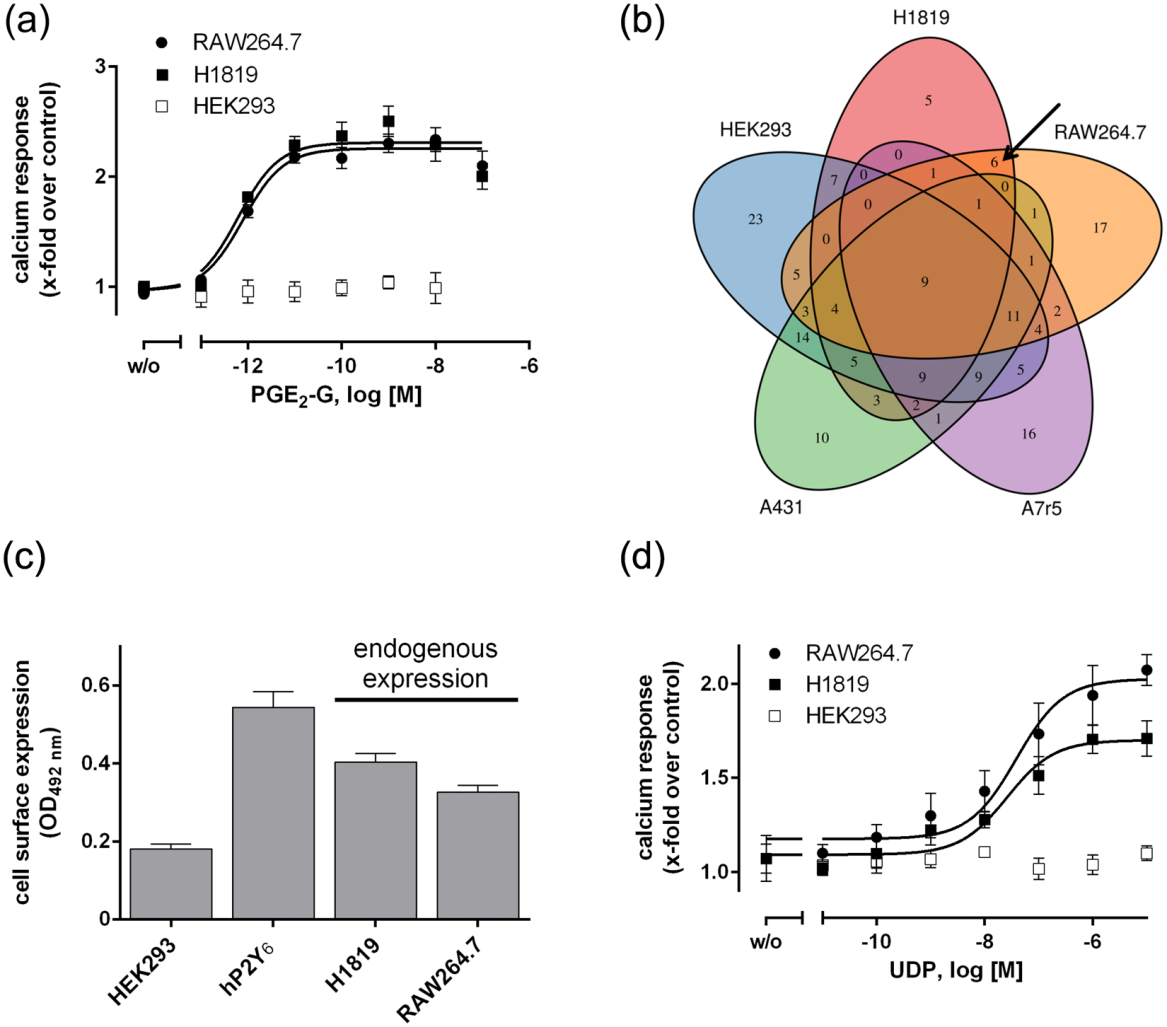
PGE2-G induces intracellular Ca2+ release in different cell lines. (**a**) Different cells lines were treated with the indicated concentrations of PGE_2_-G, and intracellular Ca^2+^ measurement was performed as described (*see Methods*). EC_50_ values were 0.8 ± 0.1 and 0.7 ± 0.1 pM for RAW264.7 and H1819 cell, respectively. Relative Fluorescence Units (RFU) for control (1% DMSO) were 1,560 ± 479, 2,106 ± 1,193, and 5,247 ± 2,016 for RAW264.7, HEK293, and H1819 cells, respectively. Maximum RFU for PGE_2_-G were 3,649 ± 678, 2,193 ± 252 and 13,143 ± 2,157 for RAW264.7, HEK293, and H1819 cells, respectively. Data are shown as RFU_max_ − RFU_min_ (ligand)/RFU_max_ − RFU_min_ (control). Data are means ± SEM of three experiments, each performed in quadruplicate. (**b**) Expression of GPCRs in the investigated cell lines. The Venn-Diagram shows the number of expressed GPCRs in the investigated cell lines. Numbers in non-overlapping regions correspond to the number of GPCRs specific for the respective cell line, whereas numbers in the overlapping areas correspond to receptors shared by two or more cell lines. 6 GPCRs were exclusively expressed in the two PGE_2_-G-responding cell lines (indicated by arrow). Receptors with an FPKM >1 were considered to be expressed. (**c**) Endogenous expression levels of P2Y_6_ in H1819 and RAW264.7 cells were determined by a cell surface ELISA using an N-terminally directed anti P2Y_6_ antibody (see *Methods*). HEK293 cells served as negative control and HEK293 cells stably transfected with hP2RY6 as positive control. Protein expression is given as optical density (OD). To determine unspecific antibody binding empty wells were treated similarly and revealed an OD_492_ nm of 0.16 ± 0.04. Data are given as means ± SEM of three independent experiments each performed in quadruplicates. (**d**) The effect of UDP on intracellular Ca^2+^ release was measured in H1819 and RAW264.7 cells (*see Methods*). Cell lines were incubated with the indicated concentrations of UDP and EC_50_ values were 38.4 ± 1.9 and 25.9 ± 2.8 nM for RAW264.7 and H1819 cells, respectively.

As seen in Fig. 1a, a Ca^2+^ mobilization assay confirms previous findings that PGE_2_-G activates its putative receptor in H1819 and RAW264.7 cells with EC_50_ values of 0.7 pM and 0.8 pM, respectively^11, 12^. PGE_2_-G had no effect on HEK293 cells. This led to the hypothesis that subtraction of all GPCRs expressed in both, PGE_2_-G-responding and -non-responding cells would provide a set of receptors that are found only in the PGE_2_-G-responder cell lines. Thus, mRNA was extracted from these cell lines and the additional PGE_2_-G-non-responding cell lines cell lines, A7r5 and A431^11^. The mRNA was subjected to RNA sequencing. The analysis revealed that a broad range of GPCRs is expressed in these cell lines (Supplementary Table S2). The number of expressed receptors above a treshold of FPKM value >1 (FPKM, fragments per kilobase of transcript per million mapped reads) was 65 (RAW264.7), 71 (A7r5), 108 (HEK293), 83 (A431), and 52 (H1819). Only 6 receptors were expressed exclusively in the PGE_2_-G-responding cell lines H1819 and RAW264.7 (Table 1, Fig. 1b). All 6 receptors are non-orphan GPCRs. GPR183, also known as the Epstein-Barr virus-induced receptor 2 (EBI2), is activated by 7α,25-dihydroxycholesterol and couples to G_i/o_ proteins^14–16^. The chemokine (C-C-motif) receptor 10 (CCR10) is activated by the chemokines CCL27 and CCL28^17^. GPR68 is known as a pH-sensing receptor and is involved in regulation of IL6-production^18^, and GPR132 can be activated by commendamide^19^. UDP is the agonist of P2Y_6_ and activation of P2Y_6_ results in generation of inositol-1,4,5-trisphosphate (IP_3_) and the subsequent release of intracellular Ca^2+^ ^20, 21^. Further, G_i_ protein-coupling was described for P2Y_6_^22^. 2-AG is the agonist of cannabinoid receptor 2 (Cnr2)^23^.

**Table 1:** GPCRs significantly expressed only in the PGE_2_-G-responding cell lines RAW264.7 and H1819.

name	gene.id (human)	FPKM					p-value
		A431	HEK293	A7r5	H1819	RAW264.7	
P2RY6	ENSG00000171631	0.18	0.29	0.9	10.09	46.64	0.005
CCR10	ENSG00000184451	0	0	0.55	7.68	3.15	0.09
CNR2	ENSG00000188822	0	0	0	1.67	2.48	0.11
GPR68	ENSG00000119714	0.51	0.36	0	1.8	9.76	0.63
GPR183	ENSG00000169508	0.14	0	0.13	1.74	39.58	1
GPR132	ENSG00000183484	0.35	0.99	0	1.55	5.69	1

The p-value is from the differential expression analysis of the human cell lines (*see Methods*).

To further prioritize the list of GPCRs for testing, we compared the expression levels (read counts) of the 6 GPCRs between the human responder and non-responder cell lines (Table 1). Only the UDP receptor P2Y_6_ showed significantly higher expression levels in the responder cell line H1819 compared to HEK293 and A431 (p = 0.005). Since our previous findings suggested that the putative PGE_2_-G receptor signals via a G_q_ and/or G_i/o_ protein^11, 12^ we focused on P2Y_6_ for further analyses.

### UDP and PGE_2_-G elevate cytosolic Ca^2+^ levels in RAW264.7 and H1819 cells

First, to validate the RNA sequencing result that P2Y_6_ is expressed in H1819 and RAW264.7 cells, we immunologically determined the receptor expression at the plasma membrane with a cell surface ELISA and measured intracellular Ca^2+^ release upon UDP stimulation. As shown in Fig. 1c, P2Y_6_ is endogenously expressed in H1819 and RAW264.7 cells but not in PGE_2_-G response-negative HEK293 cells. Further, UDP is an agonist in the Ca^2+^ assay with EC_50_ values of 38.4 ± 1.9 and 25.9 ± 2.8 nM in RAW264.7 and H1819 cells, respectively, and has no effect on HEK293 cells (Fig. 1d). The EC_50_ values were almost identical to the EC_50_ value determined in 1321N1 human astrocytoma cells stably transfected with P2RY6^24^.

**Figure 2:**
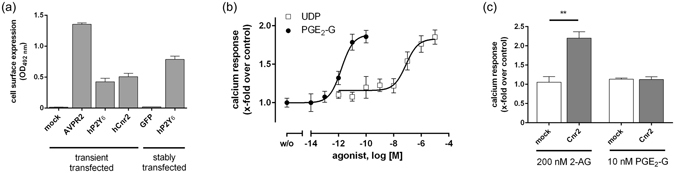
Effect of UDP and PGE2-G on transfected HEK293 cells. (**a**) HEK293 cells were transiently transfected with either HA-tagged version of hP2RY6 and hCNR2 and the expression levels of receptors were measured by a cell surface ELISA (see *Methods*). As a positive control the human V2-vasopressin receptor (hAVPR2), N-terminally tagged with an HA tag, was used. eGFP and hP2RY6_eGFP were stably transfected in HEK293 cells and cell surface expression was measured. (**b**) HEK293 cells transfected with P2RY6 were used for intracellular Ca^2+^ measurements *(see Methods*). Indicated concentrations of UDP and PGE_2_-G revealed EC_50_ values of 78.3 ± 6.7 nM and 1.2 ± 0.08 pM, respectively. (**c**) In contrast to PGE_2_-G, 200 nM of 2-AG elevated intracellular Ca^2+^ in CNR2-transfected cells. RFU for mock-transfected cells were 1,832 ± 299. All data are given as means ± SEM of three independent experiments each performed in triplicate. *p < 0.05, **p < 0.01, ***p < 0.001 (paired Student’s t test).

Next, we cloned the human P2RY6 into the mammalian expression vector pcDps to test the ability of PGE_2_-G to activate this receptor in a heterologous expression system. After transfection, the receptor was detectable at the surface of HEK293 cells (Fig. 2a). Both, PGE_2_-G and UDP increased the cytosolic Ca^2+^ levels in HEK293 cells transiently transfected with P2RY6 in a concentration-dependent manner (Fig. 2b) whereas PGE_2_-G and UDP had no effect on non-transfected cells (Fig. 1a and d). PGE_2_-G revealed the same potency (~1 pM) as observed in RAW264.7 and H1819 cells^11, 12^. Because Cnr2 is exclusively expressed in PGE_2_-G-responding cells (Table 1), and its agonist 2-AG is chemically related to PGE_2_-G, we transiently transfected CNR2 in HEK293 cells for functional testing. 2-AG induced a robust intracellular Ca^2+^ increase in these cells, but PGE_2_-G did not (Fig. 2c), excluding this receptor as potential target of PGE_2_-G.

### UDP- and PGE_2_-G-activated P2Y_6_ couples to G_q_- and G_i/o_ proteins

**Figure 3:**
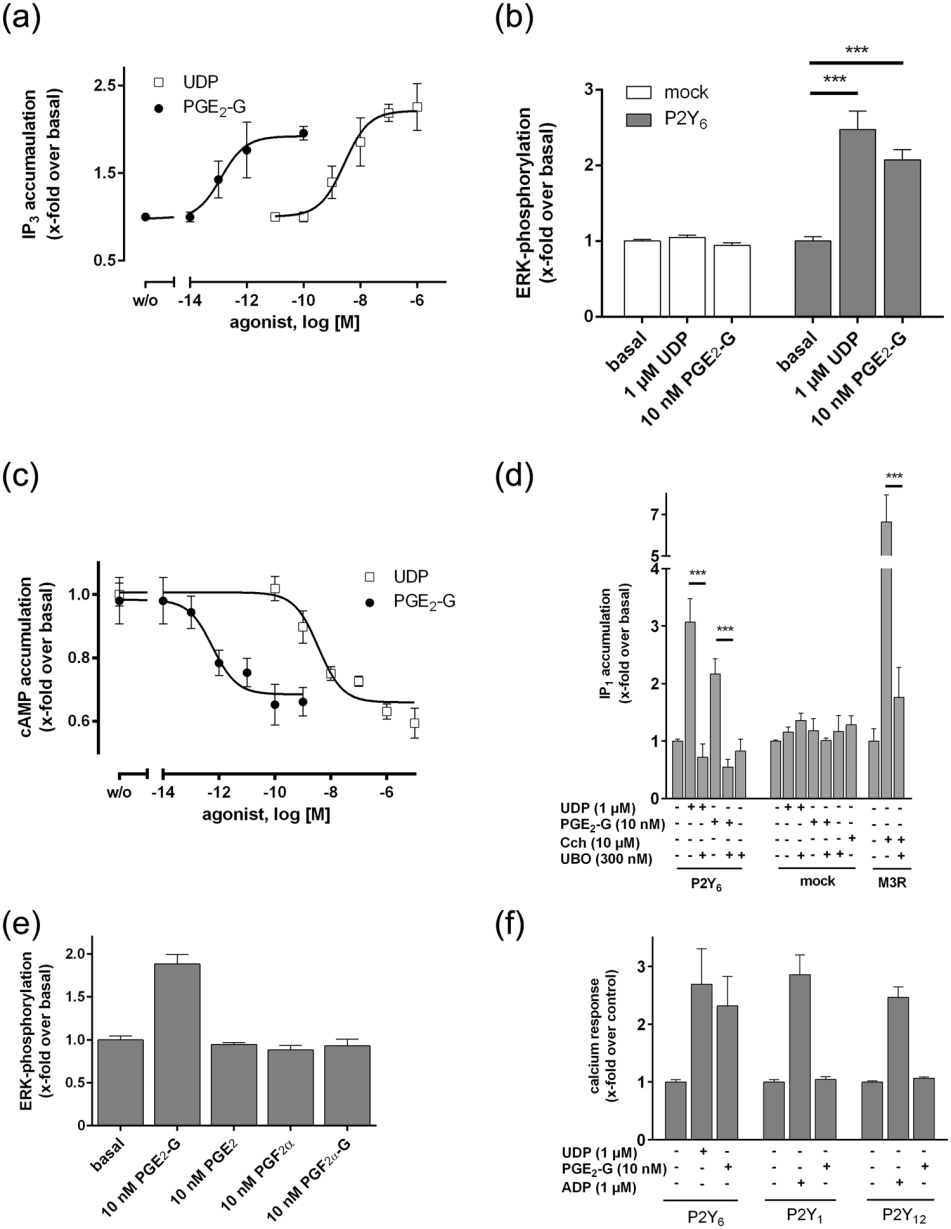
P2Y6 activated by UDP- and PGE2-G couples to Gq- and Gi/o proteins. (**a**) In an IP_3_ assay with P2RY6-transfected HEK293 cells, UDP and PGE_2_-G revealed EC_50_ values of 2.7 ± 0.1 nM and 0.2 ± 0.01 pM, respectively. (**b**) PGE_2_-G and UDP induce ERK1/2 phosphorylation. Mock- and P2RY6-transfected HEK293 cells were treated with the indicated concentrations of UDP and PGE_2_-G, and ERK phosphorylation was measured with the AlphaScreen^®^ *SureFire* ERK 1/2 assay. (**c**) In a cAMP-inhibition assay HEK293 cells transfected with P2RY6 were incubated with various concentrations of PGE_2_-G or UDP in the presence of 2.5 µM forskolin (*see Methods*). The EC_50_ values of PGE_2_-G and UDP were 0.6 ± 0.2 pM and 3.6 ± 0.3 nM, respectively. Basal cAMP levels before and after stimulation with forskolin were 2.3 ± 0.6 and 106 ± 7.3 nM/well, respectively. (**d**) HEK293 cells transfected with P2RY6 were incubated with UDP or PGE_2_-G in the presence or absence of UBO and IP_1_ accumulation assay was performed as described. As a control, the G_q_ protein-coupled muscarinic acetylcholine receptor (M3R) receptor stimulated with carbachol (Cch) was used. (**e**) HEK293 cells stably transfected with P2RY6 were incubated with indicated concentrations of prostaglandins and PG-Gs and ERK1/2 phosphorylation assay was performed as described under *Methods*. All concentrations were tested on empty vector- (mock-) transfected cells and showed no effect in the used second messenger assays. (**f)** The effect of PGE_2_-G on intracellular Ca^2+^ release was determined in HEK293 cells transfected with P2RY1 or P2RY12 (*see Methods*). All data are means ± SEM of three independent experiments, each performed in triplicate. *p < 0.05, **p < 0.01, ***p < 0.001 (paired Student’s t test).

As previously shown, PGE_2_-G induces an increase in IP_3_ levels in RAW264.7 cells^11^. We performed an IP_3_ assay in P2RY6-transfected HEK293 cells. Stimulation with UDP and PGE_2_-G increased IP_3_ levels with EC_50_ values of 2.7 ± 0.1 nM and 0.2 ± 0.01 pM, respectively, (Fig. 3a). In addition, activation by UDP and PGE_2_-G led to ERK1/2 phosphorylation (Fig. 3b) in P2RY6-transfected HEK293 cells, and both compounds suppressed cAMP formation in these cells with EC_50_ values of 0.6 ± 0.2 pM and 3.6 ± 0.3 nM for PGE_2_-G and UDP, respectively (Fig. 3c). The specificity of G_q_ coupling after PGE_2_-G activation was verified with a selective inhibitor for the G_q_ protein^25^ (UBO). UBO can significantly block the IP formation after stimulation with UDP and PGE_2_-G (Fig. 3d).

In previous studies, Nirodi and co-workers showed that PGF_2α_ led to Ca^2+^ release in RAW264.7 cells^11^. To provide evidence that the PGF_2α_ response is not caused by P2Y_6_, we tested PGF_2α_ on HEK293 cells transfected with P2RY6. As shown in Fig. 3e, PGF_2α_ had no effect on these cells. Additionally, PGF_2α_-G, and PGE_2_ showed no response in P2RY6-transfected cells (Fig. 3e). These results are consistent with previous observation that PGD_2_-G, PGF_2α_-G, and PGE_2_ had no effect on RAW264.7 cells in Ca^2+^ measurements^11^. Finally, to test whether PGE_2_-G activates other P2Y receptors, we tested P2Y_1_ and P2Y_12_ for Ca^2+^ release upon PGE_2_-G stimulation. As shown in Fig. 3f these receptors led to Ca^2+^ release after stimulation with their agonist ADP but not with PGE_2_-G. This indicates that the PGE_2_-G/P2Y_6_ pair is a highly specific endogenous signalling system.

**Figure 4:**
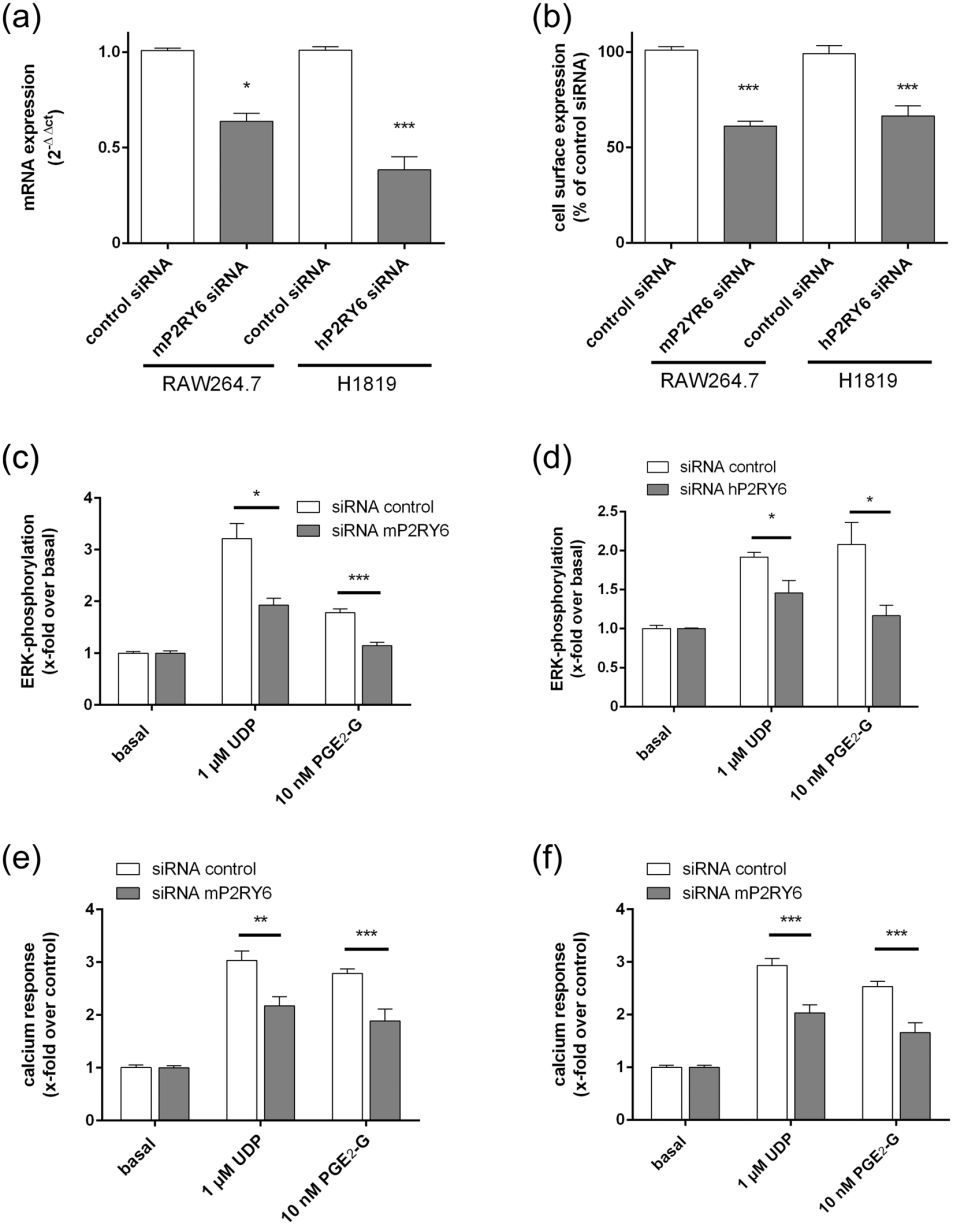
Knock-down of P2Y6 revealed decreased signal transduction of UDP and PGE2-G. (**a**) and (**b**) RAW264.7 and H1819 were transfected with siRNA for mouse and human P2RY6, respectively. **(a)** mRNA expression levels of P2RY6 were determined (*see Methods*) and were normalized to β2-microglobulin (C_t_ were 14.6 ± 0.2 and 17.4 ± 0.1 for RAW264.7 and H1819, respectively). Values are given as mean of 2^−ΔΔCt^ ± SEM and statistical analysis was performed according to ref. 58. (**b**) Protein expression levels of P2Y_6_ were determined using a cell surface ELISA using an N-terminus-directed anti-P2Y6 specific antibody (see *Methods*) and are given as OD at 492 nm. Data are given as means ± SEM of three independent experiments performed in quadruplicates. (**c**–**f**) siRNA-transfected RAW264.7 (**c**,**e**) and H1819 (**d**,**f**) were incubated with the indicated concentrations of UDP and PGE_2_-G, and ERK1/2 phosphorylation (**c**,**d**) and Ca^2+^ mobilization assays (**e**,**f**) were performed as described. All data are means ± SEM of three independent experiments, each performed in triplicate. *p < 0.05, **p < 0.01, ***p < 0.001 (paired Student’s t test).

Next, we tested whether P2Y_6_ activation is responsible for the previously reported PGE_2_-G-induced ERK1/2 phosphorylation in RAW264.7 and H1819 cells^11, 12^. We performed siRNA knock-down experiments for the mouse and human P2RY6 orthologues in RAW264.7 and H1819 cells, respectively. Knock-down of the receptor mRNA and protein expression was verified by RT-qPCR experiments and cell surface ELISA, respectively. As shown in Fig. 4a,b, a significant down-regulation of P2RY6 mRNA and P2Y_6_ protein expression was found after 48 h. The down-regulation also decreased ERK1/2 phosphorylation and Ca^2+^ release after stimulation with UDP and PGE_2_-G (Fig. 4c–f).

**Figure 5:**
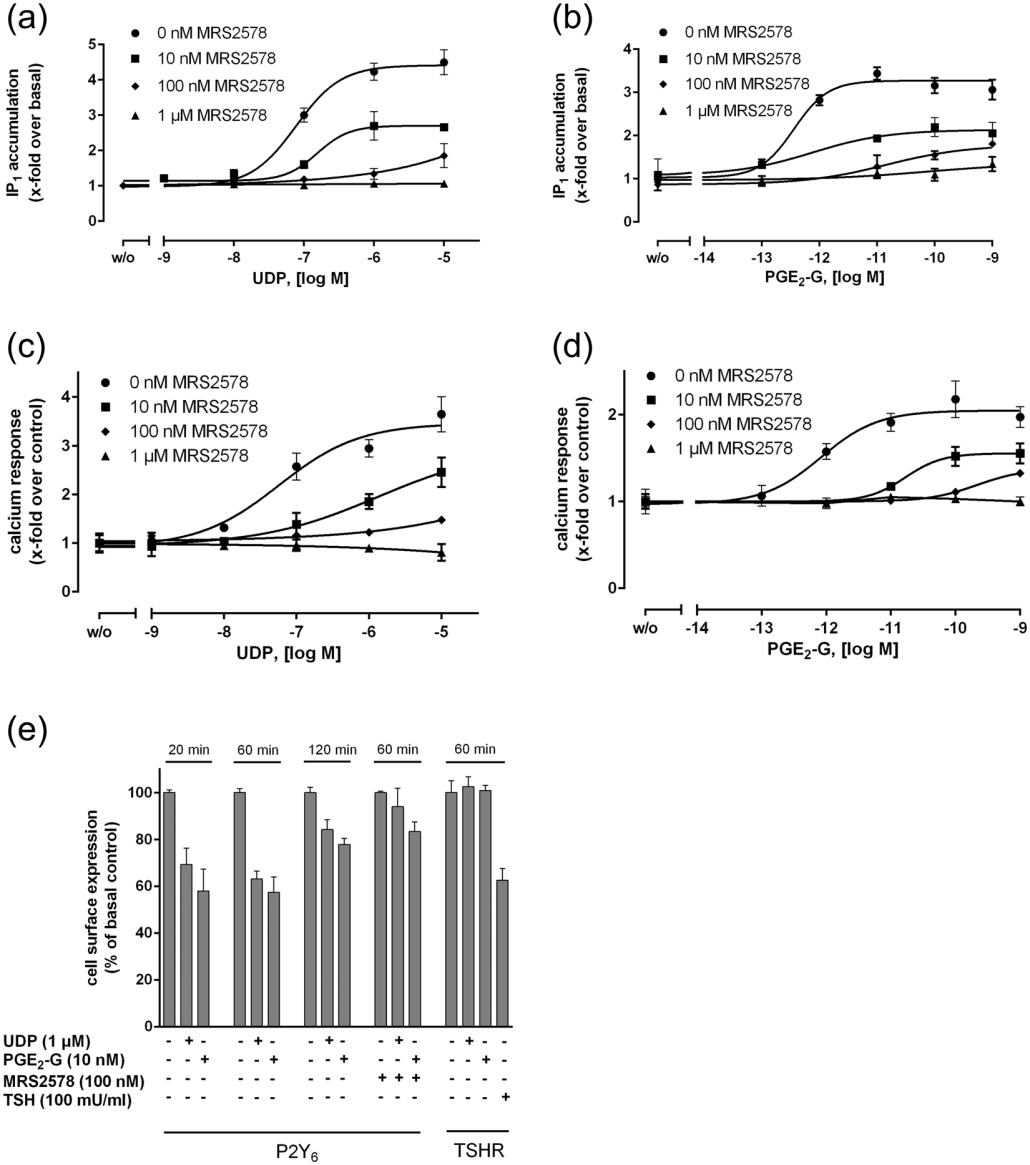
UDP- and PGE2-G-induced signaling can be blocked by the P2Y6 antagonist MRS2578. HEK293 cells were stably transfected with P2RY6, and the IP_1_ accumulation assay and measurement of Ca^2+^ release was performed as described under *Methods*. Concentration-response curves of UDP (**a**,**c**) and PGE_2_-G (**b**,**d**) were performed in the absence and presence of different concentrations of the antagonist MRS2578 in 1% DMSO. EC_50_ values for UDP and PGE_2_-G in IP_1_ accumulation assay were 77.3 ± 3.4 nM and 0.3 ± 0.02 pM, respectively, and 57.1 ± 2.5 nM and 0.8 ± 0.08 pM in Ca^2+^ mobilization assay. Data are means ± SEM of three independent experiments, each performed in triplicate. (**e**) HEK293 cells were transiently transfected with hP2RY6 and the HA-tagged human TSH receptor (TSHR) and the receptor expression levels were measured by cell surface ELISA (see *Methods*). Both, UDP and PGE_2_-G induced a time-dependent internalization of the P2Y_6_ whereas both compounds had no effect on the cell surface expression of the TSH receptor. The TSH receptor was stimulated with bovine TSH (100 mU/ml). The non-specific antibody binding to empty vector-transfected cells revealed an OD_492_ nm of 0.01 ± 0.002. Data are given as mean ± SEM of three independent experiments each performed in quadruplicates.

Increasing concentrations of the selective P2Y_6_ antagonist MRS2578^26^ reduced both the efficacies and potencies of UDP-induced IP_1_ accumulation (Fig. 5a). The different MRS2578 concentrations had a similar effect on UDP- and PGE_2_-G-triggered IP_1_ accumulation (Fig. 5b). Similarly, MRS2578 had the same effects on UDP- and PGE_2_-G-triggered Ca^2+^ release (Fig. 5c,d). Finally, we studied agonist-induced receptor internalization in the cell surface ELISA. As shown in Fig. 5e, cell surface expression levels of P2Y_6_ were reduced following UDP and PGE_2_-G stimulation and the time-dependent internalization showed no differences between the agonists. The antagonist MRS2578 blocked the internalization (Fig. 5e).

In sum these data clearly demonstrate that signal transduction of PGE_2_-G requires the presence of the UDP receptor P2Y_6_. Our results indicate that the antagonist MRS2578 has very similar effects on the function of both UDP and PGE_2_-G. This is compatible with a scenario that both agonists share the binding side but it does not prove it. However, there is also the possibility that PGE_2_-G somehow releases UDP from the cell which in an autocrine or paracrine manner activates P2Y_6_. To convincingly demonstrate that PGE_2_-G directly acts on P2Y_6_, we explored the binding sites of both agonists. The identification of determinants that are specific for binding of only one of the agonists would be highly supportive for a two-agonists-scenario at P2Y_6_.

### UDP and PGE_2_-G share the ligand binding site of P2Y_6_

**Figure 6:**
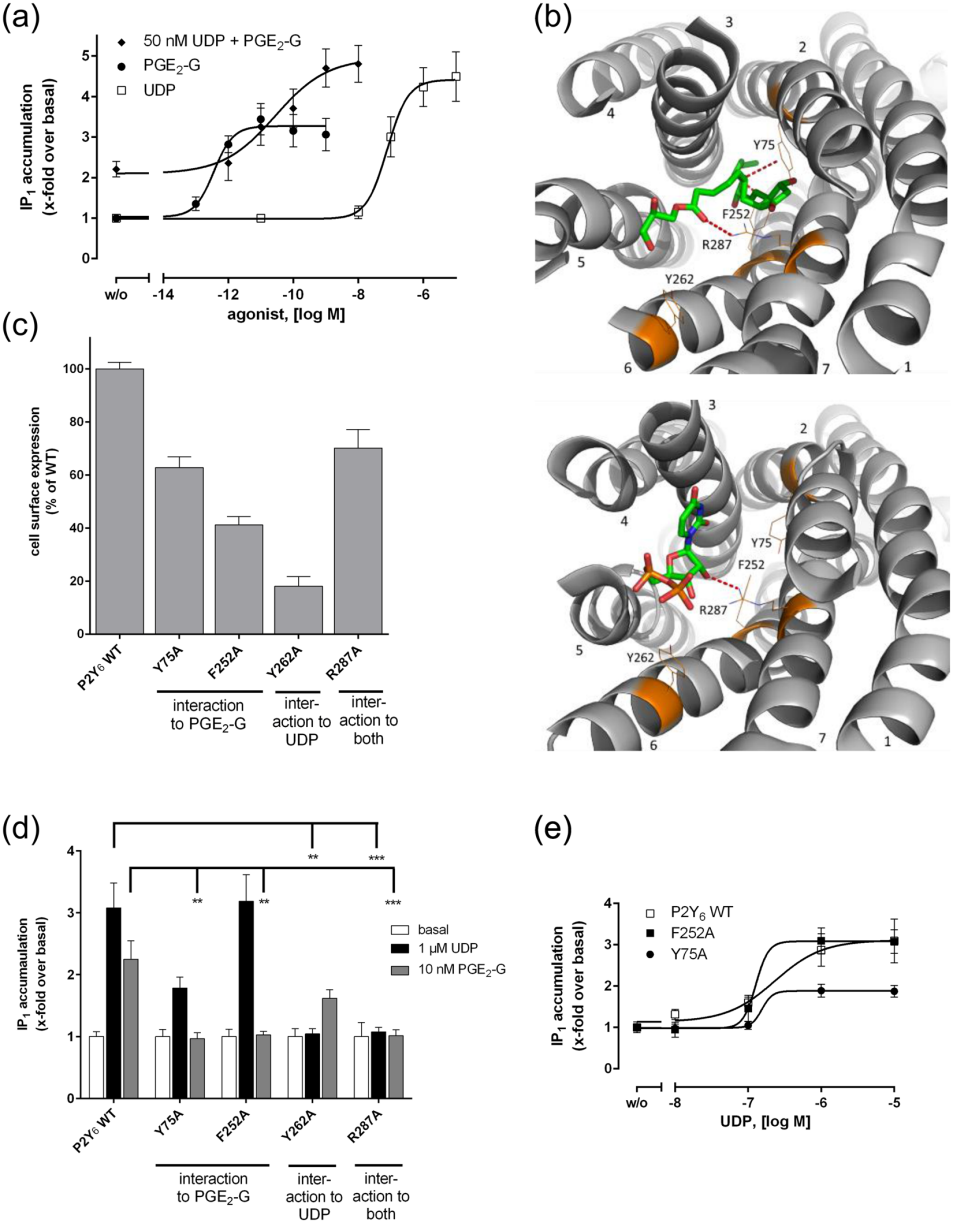
UDP and PGE2-G have overlapping agonist binding sites. (**a**) Concentration-response curves of UDP and PGE_2_-G alone and of PGE_2_-G + 50 nM UDP on HEK293 cells transfected with P2RY6 were determined in IP_1_ accumulation assays (*see Methods*). EC_50_ values for UDP and PGE_2_-G were 18.9 ± 2.3 nM and 0.3 ± 0.1 pM, respectively. (**b**) Extracellular view of the top scoring pose of PGE_2_-G (green) docked in the comparative model of the P2Y_6_ receptor (gray) (top) and top scoring pose of UDP (green) (bottom). Side chains of residues Y75, F252, Y262, and R287 are indicated as orange lines. Interactions captured in the majority of the top scoring poses are indicated as dashed red lines. Helices are numbered from N- to C-terminal. (**c**) Cell surface expression of mutant P2Y_6_ receptors was determined as described. Optical density (OD) is given as percentage of P2Y_6_ WT minus OD of mock-transfected cells. Data are given as means ± SEM of three independent experiments performed in triplicate. (**d**) and (**e**) HEK293 cells were transfected with wildtype and mutant P2RY6 and IP_1_ accumulation assays were performed as described. (**d**) Indicated concentrations of UDP and PGE_2_-G were tested on mutant P2Y_6_ receptors. (**e**) Indicated concentrations of UDP revealed EC_50_ values of 15.4 ± 0.9 nM and 12.7 ± 2.1 nM for Y75A and F252A, respectively. All data are means ± SEM of three independent experiments, each performed in triplicate. *p < 0.05, **p < 0.01, ***p < 0.001 (paired Student’s t test).

UDP is a well-established endogenous agonist for P2Y_6_^21^. Our studies above showed that application of both, UDP and PGE_2_-G, leads to activation of the human and mouse P2Y_6_. This raises the question of whether the binding sites for UDP and PGE_2_-G at P2Y_6_ overlap or are separated from each other. Unfortunately, there is no competitive antagonist specific for UDP at P2Y_6_ and, therefore, Schild plot analyses could not be properly performed to clearly answer this question. However, to experimentally approach this important question we incubated P2RY6-transfected HEK cells with a submaximal concentration of UDP and performed concentration-response curve of PGE_2_-G in an IP_1_ accumulation assays. As shown in Fig. 6a, UDP at non-saturating concentrations (50 nM) increased IP_1_ formation. Addition of increasing concentrations of PGE_2_-G further elevated IP_1_ levels but did not change the maximum response in this assay in an additive manner. EC_50_ values of UDP and PGE_2_-G alone were 77.3 ± 3.4 nM and 0.3 ± 0.02 pM, respectively (Fig. 6a). In the presence of sub-maximum UDP concentration the concentration-response curve of PGE_2_-G shifted to higher concentrations with an EC_50_ of 27.6 ± 2.8 pM. This result is difficult to interpret because it does not exclude different binding sides but it also does not directly support it (see *Discussion*).

Currently, there is no direct structural information on the P2Y_6_. However, recent advances in solving the crystal structures of other GPCRs allow for generation of a P2Y_6_ homology model and ligand docking. To estimate whether the different agonists may have similar binding properties, we simulated binding by docking the agonists into the comparative model of P2Y_6_ (Fig. 6b). The model suggested that UDP and PGE_2_-G have an overlapping binding pocket bordered by transmembrane helices (TM) 3, 6, and 7 with PGE_2_-G extending further to TM 2. The model suggests that UDP and PGE_2_-G share a number of interaction determinants but some are specific for the individual agonists. For example, UDP has distinct interactions with position R287 and Y262 whereas PGE_2_-G is orientated to position Y75, R287 and F252.

To study the functional relevance of the individual positions we performed mutagenesis studies, changing the positions individually to alanine and testing the mutants in IP_1_ accumulation assays. All mutants were expressed at the cell surface (Fig. 6c). Mutation of position Y262 to alanine displayed significantly reduced activity upon stimulation with UDP but was fully activated by PGE_2_-G (Fig. 6d). Alanine mutation of the positions Y75 and F252, predicted to interact only with PGE_2_-G, resulted in a loss of PGE_2_-G-induced IP_1_ formation, whereas UDP efficacy remained unchanged. Finally, alanine substitution of position R287 decreased receptor function for both agonists (Fig. 6d). In concentration-response experiments UDP displayed unchanged EC_50_ values at mutant receptors Y75A and F252A with 15.4 ± 0.9 nM and 12.7 ± 2.1 nM, respectively (Fig. 6e). The ability to separate the activation abilities of UDP and PGE_2_-G by distinct mutations excludes the possibility that a PGE_2_-G-induced UDP release and subsequent P2Y_6_ activation is responsible for the activity of PGE_2_-G at P2Y_6_-expressing cells.

## Discussion

Prostaglandin glycerol esters represent a separate class of prostaglandins that derive from COX-2-selective oxygenation of 2-AG. In contrast to prostaglandins, which have been extensively studied for physiological functions and receptor signalling, the function and signalling pathway(s) of PG-Gs are still unknown. Here, we describe P2Y_6_ as a physiological target of this unique group of bioactive lipids. After transient and stable expression in HEK293 cells and testing in different functional assays (see Figs 2–6) P2Y_6_ was discovered to be the G_i_/G_q_ protein–coupled receptor for PGE_2_-G. Activation of this signalling pathway resulted in reduction of intracellular cAMP levels, ERK1/2 phosphorylation, IP formation, and intracellular Ca^2+^ release similar to those found in a macrophage cell line RAW264.7 and H1819 cells (see Fig. 1a) where P2Y_6_ is highly expressed (Table 1; Fig. 1c).

Classically, agonist/GPCR pairs have been identified by screening potential targets (*e.g*., GPCR libraries) with a biologically active compound and iterative enrichment of mRNA from cells/organs that respond to this compound. The “reverse pharmacology” approach starts with tissue extracts that have an effect on a given target, *e.g*., an orphan GPCR. Using different purification and fractionation steps, the endogenous agonist can be identified within those extracts^27^. Here, we took advantage of next generation sequencing technology to compare the transcriptomes of PGE_2_-G-responding and non-responding cell lines in a subtractive approach. We used this approach not only to identify the receptors existing in PGE_2_-G-responding cells but also to provide specific controls. For example, PGE_2_-G is highly specific at P2Y_6_ because other structurally related GPCRs of the P2Y group with similar signal transduction abilities were either tested in the orphan GPCR-expressing cell line panel (see Supplementary Table S1) or were not exclusively expressed in PGE_2_-G responding cell lines (see Supplementary Table S3). Additionally, other P2Y receptors do not increase intracellular Ca^2+^ levels upon PGE_2_-G stimulation in a heterologous expression system (see Fig. 3f).

P2Y_6_ is expressed in a number of cells and tissues including the spleen, thymus, intestine, leukocytes, and aorta. Studies with P2Y_6_-deficient mice have shown that this receptor is involved in both, the direct contraction and endothelium-dependent relaxation of the aorta by UDP^28^. Its relevance in immune functions was demonstrated in P2Y_6_-deficient CD4^+^ T cells where the receptor fine-tunes the activation of T cells in allergen-induced pulmonary inflammation^29, 30^. Further, P2Y_6_ deficiency can reduce macrophage-mediated cholesterol uptake in atherosclerotic lesions^31^. One can speculate that, besides UDP, PGE_2_-G is involved in mediating these functions. However, dissecting PGE_2_-G-mediated effects from those of UDP is not trivial since specific inhibition of PGE_2_-G biosynthesis is currently not possible and COX-2 inhibition will always affect other prostaglandins and PG-Gs. Therefore, development of PGE_2_-G-specific receptor blockers or synthesis of inhibitors is required to identify the physiological contributions of PGE_2_-G and UDP to P2Y_6_-mediated signalling.

The endogenous agonists for most GPCRs have EC_50_ values >1 nM but rarely below 10 pM. With an EC_50_ value of ~1 pM the PGE_2_-G/P2Y_6_ is an extraordinarily high affinity agonist/receptor pair. This is consistent with the observation that PGE_2_-G is synthesized in low concentrations by COX-2 in macrophages and is susceptible to hydrolysis^4, 7^. Activation of P2Y_6_ would require high affinity to meet these physiological conditions. The inducible enzyme COX-2 is expressed in neurons and radial glia cells and is involved in pathophysiological responses such as inflammation and allergic responses^32^. A recent study revealed a role of COX-2-derived PGE_2_-G in inflammation and macrophage activation, further increasing IL-1β production and hyperalgesia^5^. Additionally, PGE_2_-G influences pain sensitivity^9^ and is involved in lowering intraocular pressure^33^. These observations suggest a para- and/or autocrine function of the PGE_2_-G/P2Y_6_ pair. In line with this, P2Y receptors, including P2Y_6_, are involved in inflammation, infection, and other (patho-)physiological conditions^20, 34^. As also shown by Zhang and co-workers, P2Y_6_ is highly expressed in RAW264.7 cells and is possibly involved in macrophage-associated immune function^35^. In addition, extracellular nucleotides are released in response to injury and inflammation to exert pro-inflammatory effects^36^. Cell lysis results in an immediate release of nucleotides to reach a concentrations >100 nM^37, 38^. This leads to stimulation of P2Y receptors to recruit macrophages. Similarly, PGE_2_-G could act via P2Y_6_ to regulate a fast and efficient recruitment of macrophages. We intensively addressed the possibility that PGE_2_-G acts indirectly by release of nucleotides. As shown in Fig. 3f there is no evidence of ATP and ADP release after incubation with PGE_2_-G since neither P2Y_1_ nor P2Y_12_ showed activity upon stimulation with PGE_2_-G. Further, the existence of mutations in P2Y_6_ that discriminate between UDP and PGE_2_-G excluded released UDP as cause of PGE_2_-G-triggered P2Y_6_ activation. It is rather evident that P2Y_6_ integrates different chemical signals related to cell damage. There is growing evidence that GPCRs can have more than one endogenous agonist (agonist promiscuity). This concept is well established for chemokine receptors^39^ but seems to occur also in other GPCRs. Previous studies on other P2Y receptors showed that, besides nucleotides, some paralogs and/or orthologues can be activated by aliphatic compounds like leukotrienes and phospholipids^40–44^.

MRS2578 reduced both, the efficacies and potencies of both agonists (see Fig. 5a–d) indicating that MRS2578 is an antagonist with mixed properties (competitive and non-competitive). This is compatible with both scenarios: a shared binding site but also different binding sites which is/are equally influenced by MRS2578. Since there is currently no competitive antagonist available for P2Y_6_ the questions whether UDP and PGE_2_-G share the binding pocket or bind at different sites is difficult to address experimentally. The potency difference of more than 4 orders of magnitude between both agonists already suggests different or additional agonist-receptor interaction sites within the P2Y_6_ molecule.

If UDP and PGE_2_-G share the agonist binding site one would expect receptor stimulation with an unchanged efficacy (E_max_ value) starting from increased basal IP levels (induced by the sub-maximum UDP concentration) as shown in Fig. 6a. EC_50_ values for UDP (18.9 ± 2.3 nM) and PGE_2_-G (0.3 ± 0.1 pM) showed the same ratio (~63,000) as observed in Ca^2+^ measurements in transfected HEK cells (~65,000, Fig. 2b). In the presence of sub-maximum UDP concentration (50 nM) the concentration-response curve of PGE_2_-G was shifted to higher concentrations. The interpretation of this finding is difficult. It may reflect that UDP binds at a different site than PGE_2_-G and allosterically influences the PGE_2_-G binding site. Latter scenario would assume only one active receptor conformation induced by the two agonist binding sites. However, there is strong evidence that different agonists stabilize or induce different active conformations^45, 46^. In such scenario both agonists compete for the same binding site but stabilize or induce different active conformations. This would lead to a shift in the concentration-response curve in a competitive manner as seen in Fig. 6a. The fact that in most functional assays the E_max_ values of PGE_2_-G are slightly lower compared to UDP (Figs 3a–c and 6a) may indicate different active conformations of P2Y_6_ as seen for partial agonists^47^.

To further address the questions whether UDP and PGE_2_-G share the binding pocket or bind at different sites we generated a homology model of P2Y_6_ and performed computer-aided ligand docking to predict the binding mode of both agonists. The predicted binding pockets of UDP and PGE_2_-G revealed shared but also specific determinants for ligand orientation. Mutation of these residues to alanine and experimental testing of these mutants (Fig. 6d,e) supported our hypothesis that UDP and PGE_2_-G most probably share interaction partners but additional determinants specific for each agonist contribute to the individual binding pockets.

In sum, we identified P2Y_6_ as the GPCR for a COX-2-selective signal transduction pathway mediated by PGE_2_-G. P2Y_6_ integrates different chemical signals to a common intracellular response. Therefore, the indirect inactivation of the PGE_2_-G/P2Y_6_ signalling system by COX-2 inhibition most likely contributes to the pharmacological effects of nonsteroidal anti-inflammatory drugs such as ibuprofen and mefenamic acid^48^.

## Methods

### Materials

If not stated otherwise, all chemicals were purchased from Sigma-Aldrich (Germany), and cell culture materials were provided by Life Technologies GmbH (Germany). FR900359 (UBO) was isolated and purified from the dried leaves of the evergreen plant *Ardisia crenata* using methanol (MeOH) extraction as described previously in detail^25^.

### Screening of orphan GPCRs with PGE_2_-G

For screening orphan GPCRs as possible targets for PGE_2_-G, the PathHunter^®^ β-Arrestin assay (DiscoveRx Co., USA) was used. It monitors the activation of a GPCR by utilizing an enzyme fragment complementation assay with β-galactosidase as the functional reporter. Briefly, the enzyme is split into two complementary portions expressed as fusion proteins in the cell. The enzyme acceptor is fused to β_2_-arrestin, and the ProLink donor peptide is fused to the GPCR of interest. Upon GPCR stimulation, β_2_-arrestin is recruited to the receptor, bringing the two fragments of β-galactosidase together. This generates an active enzyme that can convert a chemiluminescent substrate, generating a signal detectable on a standard microplate reader (see manufacturer’s instructions). In total, 78 GPCRs, previously considered orphan and individually transfected into cells, were screened by DiscoveRx (Supplementary Table S1). The percentage activity given in the results is calculated using the following formula: % activity = 100% × (mean RLU of test sample - mean RLU of vehicle control)/mean RLU of vehicle control).

### Library construction and RNA sequencing

RAW264.7, H1819, A7r5, and HEK293 cells were purchased from ATCC^®^. A431 cells were a gift of Dr Stanley Cohen (Vanderbilt University, USA). Total RNA from RAW264.7, HEK293, A7r5, H1819, and A413 cells was isolated using TRI REAGENT™ (Sigma-Aldrich) according to the manufacturer’s instructions. RNA quantity was measured with a spectrometer (Nanodrop ND 1000), and RNA quality was analysed on the Agilent 2100 Bioanalyzer using the RNA 6000 Nano Chip (Agilent Technologies, USA). We only included RNA samples with an RIN value above 8. Indexed cDNA libraries were generated using TruSeq RNA Sample Preparation kits v2 (Illumina, USA) according to the manufacturer’s protocol. The average library size was 300 bp as determined on the Agilent 2100 Bioanalyzer with DNA 1000 Chips.

The libraries were sequenced on the Illumina HiScanSQ Sequencing System (Interdisciplinary Centre for Clinical Research, Leipzig), generating on average 11.8 ± 1.5 million 101-bp raw paired-end reads per sample on one flow cell lane.

### Gene quantification and differential expression analysis

After intensities call, raw reads were separated according to library indices allowing up to one mismatch in the index sequence, but requiring that all bases have a quality score above 15 (PHRED-scale). After assigning reads to samples, we used an in-house sequence analysing pipeline to trim the adapters and remove reads that were shorter than 60 bp or had more than five bases with a quality score below 15 (PHRED-scale). Reads were mapped to the reference genome of human (February 2009 GRCh37/hg19), mouse (July 2007 NCBI37/mm9), and rat (November 2004 rno4), respectively, using Tophat 2.0.6.^49^, which aligns reads using Bowtie2 v2.1.0. Reads that did not map uniquely to a genome position were excluded. Running a differential expression analysis across species boundaries is not a straightforward task, since the comparison would have to address issues like changes in gene structure, gene duplications, and deletions. Thus, we combined a differential expression analysis of the human cell lines with a general GPCR expression profile of human, mouse, and rat cell lines. To assess GPCR expression, we combined information from the HUGO gene nomenclature committee (HGNC)^50^ and the EMBL-EBI InterPro database^51^ to retrieve a list of human GPCRs. Afterwards, the expression levels of these receptors in the respective cell lines, as well as their orthologues from mouse and rat (retrieved via BioMart^52^ from Ensembl v82), were obtained as FPKM by using Cufflinks v2.1.1^53^. A receptor with an FPKM >1 was considered to be expressed. For the differential expression analysis, the transcript level for each gene was obtained as read count by intersecting mapping results with gene annotations using BEDTools IntersectBed^54^. Using the DESeq software package^55^, differential expression of genes between the positive human cell line H1819 and the negative human cell lines HEK293 and A431 was examined. Differentially expressed genes with a p-value < 0.05 were considered as statistically significant.

### Generation of receptor constructs

cDNA from H1819 cells was used to amplify and clone the P2RY6 and CNR2 coding sequences. They were double-tagged with an N-terminal HA epitope and a C-terminal FLAG epitope and, for transient transfection, introduced into the mammalian expression vector pcDps^56^. All mutant constructs were generated by a PCR-based site-directed mutagenesis and fragment replacement strategy. For stable transfection, P2RY6 was sub-cloned into the pIRES-eGFP vector (CLONTECH Laboratories, USA). All constructs were verified by sequencing.

### Cell culture and transfection

RAW264.7 cells were grown in DMEM supplemented with 10% FBS, A7r5 (rat fibroblast) cells in DMEM with 10% FBS, and A431 (human squamous carcinoma) cells in RPMI supplemented with 10% FBS. All cell lines were grown at 37 °C in a humidified 5% CO_2_ incubator. For functional assays, receptor constructs were heterologously expressed in human embryonic kidney (HEK293) cells upon transient or stable transfection. Cells were grown in DMEM/F12 supplemented with 10% FBS, 100 units/ml penicillin, and 100 µg/ml streptomycin. For the cAMP-inhibition assay, ERK1/2 phosphorylation assay, and Ca^2+^ mobilization assay, cells were split into 96-well plates (2.0 × 10^4^ cells/well) and transfected with 250 ng vector construct using MACSfectin™ (Miltenyi Biotec, Germany) according to manufacturer’s protocol. Empty vector (mock) served as the negative control. For siRNA experiments, cells were seeded in 6-well plates (4.0 × 10^5^ and 6.0 × 10^5^ cells/well for RAW264.7 and H1819, respectively). RAW264.7 and H1819 cells were transfected with 150 pMol of mP2RY6 siRNA and hP2RY6 siRNA (Santa Cruz Biotechnology, USA), respectively, using Viromer^®^ Blue (Lipocalyx, Germany). As a negative control, control siRNA-A (Santa Cruz Biotechnology, USA) was used. FITC-labelled siRNA served as a transfection control.

To generate a cell line stably expressing P2Y_6_, HEK293 cells were seeded in 6-well plates (7.5 × 10^5^ cells/well) and transfected with 3 µg vector using MACSfectin™ as transfection regent according to the manufacturer’s protocol. The pIRES-eGFP vector served as negative control. Stably transfected HEK293 cells were cultured in the presence of geneticin (500 µg/ml) for selection.

To estimate cell surface expression of heterologously expressed receptors carrying an N-terminal HA tag, an indirect cellular ELISA was used^57^. To determine the endogenous cell surface expression of P2Y_6_, the same ELISA procedure was performed with minor modifications. Briefly, after 4% paraformaldehyde fixation and blocking with 10% FBS, cells were incubated with the primary anti-P2Y_6_ antibody (sc-15215; Santa Cruz Biotechnology, USA) in 1:1,000 dilution for 1 h. After washing with PBS, cells were incubated with peroxidase-conjugated secondary antibody anti-goat IgG (sc-2020, Santa Cruz Biotechnology, USA) in 1:5,000 dilutions.

### Intracellular Ca^2+^ measurement

For fluorometric measurements of intracellular Ca^2+^ levels with the FlexStationII instrument (Molecular Devices), RAW264.7 and H1819 cells were seeded into 96-well plates (3.0 × 10^4^ cells/well) 24 h prior to assay. Ca^2+^ measurements with transiently transfected HEK293 cells were performed 48 h after transfection. Cells were loaded with 200 µl Calcium 5 reagent (Explorer Kit, Molecular Devices, USA) for 60 min at 37 °C, and the assay was performed as described^11^. Agonists and inhibitors were solved in DMSO (100x) and diluted 1:20 in 96-well compound plates containing HBSS. 50 µl of compound solution were added to the assay plate resulting in final concentration of 1% DMSO. Fold-response was calculated by RFU_max_ − RFU_min_ (ligand)/RFU_max_ − RFU_min_ (vehicle). EC_50_ values were calculated by using GraphPad Prism6 software (GraphPad, USA).

### Measurement of ERK-phosphorylation

Cells were transferred to serum-free medium 2 h prior to assay. Ligands and controls (10 µl of 10× concentrates) were added to 90 µl medium and incubated for 5 min at 37 °C. The final concentration of DMSO was 0.2%. The reaction was stopped by aspiration of the medium and addition of 50 µl lysis buffer (PerkinElmer Life Sciences). To measure ERK1/2 phosphorylation, the AlphaScreen^®^ *SureFire* ERK 1/2 assay kit (PerkinElmer Life Sciences) was used with the high sensitivity protocol. Phosphorylated ERK1/2 was measured in 384-well white OptiPlate microplates (PerkinElmer Life Sciences) with the Fusion AlphaScreen multilabel reader (PerkinElmer Life Sciences).

### cAMP-inhibition assay

After transfection (72 h), cells were washed once with DMEM/F12 containing 1 mM 3-isobutyl-methyl-xanthine (IBMX) followed by incubation in the presence of the indicated compounds and forskolin (2.5 µM) for 15 min at 37 °C. The final concentration of DMSO was 1%. Cells were lysed in 25 μl lysis buffer (5 mM HEPES; 0.1% BSA; 0.3% Tween20; 1 mM IBMX; pH 7.4) and kept frozen at −20 °C until measurement. To measure cAMP concentration, the AlphaScreen cAMP assay kit (PerkinElmer Life Sciences) was used according to the manufacturer’s protocol.

### siRNA experiments and RT-qPCR

After transfection (24 h), cells were harvested and seeded overnight into 96-well plates (3.0 × 10^4^ H1819 cells/well and 2.5 × 10^4^ RAW264.7 cells/well) and 6-well plates (4.0 × 10^5^ cells/well) for functional assays and RNA isolation, respectively. Measurement of ERK1/2 phosphorylation and Ca^2+^ release was performed as described above. For analysis of receptor’s mRNA expression after siRNA transfection (see above), RNA from cells was isolated using TRI REAGENT™ (Sigma-Aldrich) according to the manufacturer’s instructions. For quantitative real-time PCR analysis (qPCR), 1 µg of total RNA was reverse-transcribed (Omniscript; Qiagen, Germany) using a mixture of oligo(dT) and random hexamer primers. qPCR was performed by GoTaq^®^ qPCR Master Mix (Promega Corporation, USA). cDNA from 25 ng total RNA and 0.2 µM forward and reverse primers was used. Oligonucleotide primers were: hP2RY6 5′-gaaccatggctttggaagg-3′ and 5′-ctgtgccattgtcccattc-3′, mP2RY6 5′-ctctctgtcctggacccaac-3′ and 5′-tgtcctgctccataactgcc-3′. The primers were designed to flank intron sequences. PCR was performed in an MX 3000 P instrument (Stratagene, USA) using the following protocol: 5 min 50 °C, 2 min 95 °C, and 40 cycles of 15 s 95 °C, 30 s 60 °C. To confirm the presence of a single amplicon, product melting curves were recorded. Threshold cycle (C_t_) values were set within the exponential phase of the PCR. Data were normalized to human or mouse β2-microglobulin and ΔC_T_ values were used to calculate the relative expression levels. Gene regulation was statistically evaluated by the 2^−ΔΔCt^ method^58^.

### Measurement of intracellular inositol phosphates

To measure intracellular IP_3_ the HitHunter^®^ Inositol (1,4,5) Triphosphate Assay (DiscoveRx, USA) was used according to manufacturer′s protocol. HEK293 cells stably transfected with hP2RY6 were seeded in a 384-well Black microtiter plate (15,000 cells/well) (Greiner Bio One, Germany) and incubated with indicated concentrations of UDP and PGE_2_-G for 20 seconds. Reaction was stopped by adding 5 µl 0.2 N perchloric acid and measurement of IP_3_ was performed with the Fusion AlphaScreen multilabel reader (PerkinElmer Life Sciences) according to manufacturer′s protocol. To measure IP_1_, HEK293 cells expressing wildtype and mutant P2Y_6_ were seeded into 384-well plates (5,000 cells/well) 24 h prior assay. After aspiration of the medium, cells were incubated with indicated concentrations of agonists/antagonist for 1 h. IP_1_ measurements using the IP-one HTFR^®^ assay kit (Cisbio assays, USA) were performed according to manufacturer′s protocol. The assays were performed with a final concentration of 1% DMSO.

### Generation of a P2Y_6_ comparative model and ligand docking

A comparative model of P2Y_6_ was constructed using the protein structure prediction software package, ROSETTA version 3^59–61^. The X-ray crystal structures of P2Y_1_ and P2Y_12_ (Protein Data Bank ID: 4xnw, 4ntj)^62–64^ were chosen as main templates based on high similarity to P2Y_6_ (e-value of 3e^−15^ with a sequence coverage of 90%) according to a search using NCBI BLASTP on sequences from the Protein Data Bank (PDB). To increase conformational sampling, these templates were supplemented with rhodopsin (1u19,2 × 72), β2-AR (2rh1,3sn6), β1-AR (2vt4,2y03), A2A (3eml,3qak), CXCR4 (3odu), D3 (3pbl), H1 (3rze), M2 (3uon), S1P1 (3v2w), M3 (4daj), κ-OR (4djh), μ-OR (4dkl), N/OFQ (4ea3), δ-OR, (4ej4), 5HT-1B (4iar), and 5HT-2B (4ib4). An initial sequence alignment of twelve P2Y receptors was performed using clustalw^65^ and a profile alignment of the GPCR templates was performed using MUSTANG^66^. Finally, a profile-profile alignment was performed using clustalw and adjustments were made to ensure that all secondary structure elements were properly aligned while moving significant gaps to loop regions. To ease computational demands, the first 15 and last 12 residues of the P2Y_6_ sequence were truncated.

After assigning coordinates to P2Y_6_ residues from each template alignment using Rosetta’s partial-thread application, RosettaCM^67^ ‘hybridizer’ was used to combine segments across all templates in an iterative Monte Carlo approach to arrive at energetically favorable compositions. In brief, RosettaCM exchanges template fragments into a starting model to achieve energetically favorable hybrid template models. Any residues still lacking coordinates were modeled de novo using 3mer and 9mer fragments. Transmembrane segments, as predicted using OCTOPUS^68^, were modeled within Rosetta’s implicit membrane potential^69^. In total, 32,000 all-atom models were generated. The resulting full sequence models were subjected to eight iterative cycles of side chain repacking and gradient minimization of ϕ, ψ, and χ angles within the membrane potential. P2Y_6_, P2Y_1_, and P2Y_12_ share a conserved disulfide bond between the N-terminal C18 and C273 in extracellular loop 3^70^. Residue pair constraints were introduced between these residues as well as C99 and C177. The top 50% of all relaxed generated models by pose score were clustered by RMSD using BCL::Cluster^71^ with a node similarity of 4 Å. The top scoring models from the three largest clusters were collected along with the top scoring models overall. Following visual inspection, a final set of 14 models were selected for docking.

Ligand docking into the comparative model of P2Y_6_ with UDP and PGE_2_-G was performed with Rosetta Ligand^72, 73^. One hundred conformations of PGE_2_-G and thirteen conformations of UDP were generated with BCL::Conf ^74^. This application builds small molecule conformations from active substructures seen in experimentally elucidated structures. For both ligands, a starting position was selected based on the average position of ligands present in all GPCR templates. The docking protocol included a low resolution (centroid mode) phase consisting of 50 cycles of 4 Å translation search and 500 cycles of 360° rotation search and a high resolution phase consisting of six cycles of side chain refinement. This phase finds an energetically favorable pose by combining minor ligand conformational flexibility with side chain refinement simultaneously. For each ligand, 12,000 poses were generated in the first round of docking. The top 50 models by interface_delta score were collected for each ligand and a second round of docking was performed beginning with each selected pose. For the second round of docking, the translation and rotation searchers were reduced to 2 Å and 180° respectively. A third focused round was performed from the same selection scheme with 1 Å and 90° search. All ligand poses generated in the third round of focused docking were clustered using BCL::Cluster and a final ensemble of 10 models for each ligand were selected based on cluster size and interface_delta. Because the docking runs did not converge on a single conformation for either ligand, all poses within the top scoring ensembles were considered for contact analysis. For each ensemble pose, the change in free energy with and without ligands bound to P2Y_6_ was calculated for each residue in the receptor. Residues with the greatest difference in predicted energy across the majority of ensemble models are suggested to be important for ligand interaction (Supplementary figure S1).

## Electronic supplementary material

Supplemental tables and figures
