# Supplementary material for: Prostaglandin E2 glyceryl ester is an endogenous agonist of the nucleotide receptor P2Y6
Source: Sci Rep. 2017 May 24;7:2380. doi: 10.1038/s41598-017-02414-8 (PMC5443783; doi:10.1038/s41598-017-02414-8)
Supplement: Supplementary file 1 — Supplemental tables and figures [file 41598_2017_2414_MOESM1_ESM.pdf]

Supplemental Data for

Prostaglandin E_2 glyceryl ester is an endogenous agonist of the nucleotide receptor $P2Y_6$

Antje Brüser[1+], Anne Zimmermann[1+], Brenda C. Crews[2], Gregory Sliwoski[5], Jens Meiler[3], Gabriele M. König[4], Evi Kostenis[4], Vera Lede[1], Lawrence J. Marnett[2], Torsten Schöneberg[1*]

[1]Rudolf Schönheimer Institute of Biochemistry, Medical Faculty, University of Leipzig, 04103 Leipzig, Germany

[2]Department of Biochemistry, Chemistry and Pharmacology, Vanderbilt Institute of Chemical Biology, Vanderbilt-Ingram Cancer Center, Vanderbilt University School of Medicine, Nashville, TN 37232-0146, USA

[3]Department of Chemistry, Center for Structural Biology, Vanderbilt University, Nashville, TN 37232-8725, USA

[4]Institute of Pharmaceutical Biology, University of Bonn, 53155 Bonn, Germany Leipzig, Germany

[5]Department of Biomedical Informatics, Vanderbilt University School of Medicine, Nashville, TN 37232-8725, USA

[+]These authors contributed equally to this work

*To whom correspondence should be addressed: Torsten Schöneberg, Institute of Biochemistry, Molecular Biochemistry, Medical Faculty, University of Leipzig, Johannisallee 30, 04103 Leipzig, Germany, Phone: +49-341-9722-176, Fax: +49-341-9722-159, E-mail: schoberg@medizin.uni-leipzig.de

This file includes:

Supplemental Table S1-3

Supplemental Figure S1

Supplemental Table S1: List of tested receptors in the Path-Hunter® biosensor Orphan GPCR cell line panel

GPCR ID	Compound ID	Assay Mode	Test Conc (μM)	Mean RLU	% Activity
ADMR	PGE2-SA	Agonist	0.001	514420	1%
BAI1	PGE2-SA	Agonist	0.001	864280	2%
BAI2	PGE2-SA	Agonist	0.001	15400	1%
BAI3	PGE2-SA	Agonist	0.001	21960	14%
CCRL2	PGE2-SA	Agonist	0.001	8400	-4%
CMKLR2	PGE2-SA	Agonist	0.001	17240	-5%
DARC	PGE2-SA	Agonist	0.001	105120	2%
EBI2	PGE2-SA	Agonist	0.001	46540	-1%
GHSR1B	PGE2-SA	Agonist	0.001	2580	-21%
GPR101	PGE2-SA	Agonist	0.001	30940	-2%
GPR103	PGE2-SA	Agonist	0.001	56020	15%
GPR107	PGE2-SA	Agonist	0.001	304860	1%
GPR12	PGE2-SA	Agonist	0.001	680	-13%
GPR123	PGE2-SA	Agonist	0.001	1221720	-1%
GPR132	PGE2-SA	Agonist	0.001	620480	-5%
GPR135	PGE2-SA	Agonist	0.001	16680	6%
GPR139	PGE2-SA	Agonist	0.001	307380	-20%
GPR141	PGE2-SA	Agonist	0.001	30420	4%
GPR142	PGE2-SA	Agonist	0.001	2660	-14%
GPR143	PGE2-SA	Agonist	0.001	91680	2%
GPR146	PGE2-SA	Agonist	0.001	15800	-2%
GPR148	PGE2-SA	Agonist	0.001	3640	-9%
GPR149	PGE2-SA	Agonist	0.001	29340	-4%
GPR15	PGE2-SA	Agonist	0.001	9440	-5%
GPR150	PGE2-SA	Agonist	0.001	31360	12%
GPR151	PGE2-SA	Agonist	0.001	57420	-2%
GPR152	PGE2-SA	Agonist	0.001	178980	-3%
GPR157	PGE2-SA	Agonist	0.001	356620	4%
GPR161	PGE2-SA	Agonist	0.001	20300	-5%
GPR162	PGE2-SA	Agonist	0.001	26360	-14%
GPR17	PGE2-SA	Agonist	0.001	805440	1%
GPR171	PGE2-SA	Agonist	0.001	4880	8%
GPR173	PGE2-SA	Agonist	0.001	62220	-10%
GPR176	PGE2-SA	Agonist	0.001	380580	3%
GPR18	PGE2-SA	Agonist	0.001	520	-19%
GPR182	PGE2-SA	Agonist	0.001	899260	9%
GPR20	PGE2-SA	Agonist	0.001	74500	-7%
GPR23	PGE2-SA	Agonist	0.001	990600	-1%
GPR25	PGE2-SA	Agonist	0.001	6140	7%
GPR26	PGE2-SA	Agonist	0.001	2200	-6%
GPR27	PGE2-SA	Agonist	0.001	219760	2%
GPR3	PGE2-SA	Agonist	0.001	1524920	-4%
GPR30	PGE2-SA	Agonist	0.001	8540	-10%
GPR31	PGE2-SA	Agonist	0.001	7680	22%
GPR32	PGE2-SA	Agonist	0.001	129240	-2%
GPR37	PGE2-SA	Agonist	0.001	822100	0%
GPR37L1	PGE2-SA	Agonist	0.001	36440	-12%

Supplemental Table S2: Expressed GPCRs in the tested cell lines

human.gene.id	rat.gene.id	mouse.gene.id	Human name	HEK293	A431	H1819	A7r5	RAW264.7
ENSG00000006638	ENSRNOG00000020585	ENSMUSG00000034881	TBXA2R	0.52	0.00	0.00	1.33	0.00
ENSG00000006831	ENSRNOG00000007990	ENSMUSG00000030168	ADIPOR2	41.35	52.62	65.43	24.11	27.71
ENSG00000008300	ENSRNOG00000034005	ENSMUSG00000023473	CELSR3	0.91	1.92	0.00	0.62	0.07
ENSG00000013588	ENSRNOG00000008412	ENSMUSG00000046733	GPRC5A	31.86	60.46	565.46	21.23	0.00
ENSG00000020181	ENSRNOG00000012991	ENSMUSG00000031486	ADGRA2	1.81	0.00	0.00	50.71	0.11
ENSG00000043591	ENSRNOG00000017002	ENSMUSG00000035283	ADRB1	0.34	0.00	14.69	0.00	0.00
ENSG00000054277	ENSRNOG00000003778	ENSMUSG00000026525	OPN3	1.31	4.96	2.22	1.24	3.82
ENSG00000064115	ENSRNOG00000001817	ENSMUSG00000040234	TM7SF3	49.96	51.53	50.18	0.74	14.77
ENSG00000064547	ENSRNOG00000010758	ENSMUSG00000031861	LPAR2	3.54	5.80	0.00	0.00	0.28
ENSG00000064989	ENSRNOG00000004622	ENSMUSG00000059588	CALCRL	0.31	1.03	0.00	0.00	12.35
ENSG00000065325	ENSRNOG00000003683	ENSMUSG00000049928	GLP2R	1.63	0.00	0.00	0.00	0.00
ENSG00000069696	ENSRNOG00000017927	ENSMUSG00000025496	DRD4	0.94	1.29	0.28	0.00	0.00
ENSG00000072071	ENSRNOG00000029134	ENSMUSG00000013033	ADGRL1	5.86	6.79	0.00	1.36	14.69
ENSG00000075073	ENSRNOG00000050658	ENSMUSG00000020081	TACR2	0.12	0.00	3.18	NA#	0.00
ENSG00000075275	ENSRNOG00000021285	ENSMUSG00000016028	CELSR1	12.93	18.77	0.00	0.00	0.00

ENSG00000077585	ENSRNOG00000002480	ENSMUSG00000021306	GPR137B	18.26	5.91	11.04	8.16	63.65
ENSG00000100739	ENSRNOG00000004488	ENSMUSG00000041347	BDKRB1	0.00	0.40	0.60	5.93	0.00
ENSG00000101850	ENSRNOG00000003543	ENSMUSG00000025333	GPR143	1.03	0.00	0.00	0.00	0.00
ENSG00000102195	ENSRNOG00000011335	ENSMUSG00000056380	GPR50	41.09	0.00	0.00	0.00	0.34
ENSG00000102468	ENSRNOG00000010063	ENSMUSG00000034997	HTR2A	0.00	0.00	0.13	3.75	0.00
ENSG00000104290	ENSRNOG00000047211	ENSMUSG00000007989	FZD3	6.45	4.76	0.00	NA#	0.00
ENSG00000104332	ENSRNOG00000017783	ENSMUSG00000031548	SFRP1	1.69	0.00	0.00	0.00	0.00
ENSG00000106113	ENSRNOG00000011145	ENSMUSG00000003476	CRHR2	0.00	0.00	0.00	22.91	0.00
ENSG00000107317	ENSRNOG00000015550	ENSMUSG00000015090	PTGDS	0.00	0.00	0.00	2.96	0.00
ENSG00000110148	ENSRNOG00000017679	ENSMUSG00000030898	CCKBR	4.35	0.00	0.00	0.00	0.00
ENSG00000111291	ENSRNOG00000008439	ENSMUSG00000030205	GPRC5D	0.00	0.00	6.23	0.14	0.00
ENSG00000112218	ENSRNOG00000007675	ENSMUSG00000040372	GPR63	4.57	0.93	0.00	0.77	0.00
ENSG00000112414	ENSRNOG00000011411	ENSMUSG00000039116	ADGRG6	18.28	15.87	0.00	9.77	0.00
ENSG00000113389	ENSRNOG00000019184	ENSMUSG00000022206	NPR3	0.23	0.00	0.00	9.93	0.00
ENSG00000114812	ENSRNOG00000042405	ENSMUSG00000032528	VIPR1	3.27	0.54	0.00	0.00	1.49
ENSG00000116678	ENSRNOG00000023664	ENSMUSG00000057722	LEPR	2.51	0.71	0.97	29.61	0.00

ENSG00000116729	ENSRNOG00000036816	ENSMUSG00000028173	WLS	60.82	75.97	66.28	244.11	13.97
ENSG00000117114	ENSRNOG00000032660	ENSMUSG00000028184	ADGRL2	51.21	5.42	88.13	51.45	0.21
ENSG00000117262	ENSRNOG00000000095	ENSMUSG00000028096	GPR89A	5.86	5.20	14.99	15.02	29.61
ENSG00000119714	ENSMUSG00000047415	ENSMUSG00000047415	GPR68	0.36	0.51	1.80	0.00	9.76
ENSG00000119737	ENSRNOG00000022176	ENSMUSG00000043999	GPR75	3.61	1.14	0.00	0.00	0.00
ENSG00000120088	ENSRNOG00000004900	ENSMUSG00000018634	CRHR1	2.87	0.00	5.24	0.00	0.00
ENSG00000121753	ENSRNOG00000014375	ENSMUSG00000028782	ADGRB2	8.59	0.22	16.39	0.00	0.00
ENSG00000121807	ENSRNOG00000049115	ENSMUSG00000079227	CCR2	0.00	0.00	0.00	0.00	0.00
ENSG00000121807	ENSRNOG00000049115	ENSMUSG00000049103	CCR2	0.00	0.00	0.00	0.00	1.18
ENSG00000121966	ENSRNOG00000003866	ENSMUSG00000045382	CXCR4	28.60	0.00	0.00	0.00	43.97
ENSG00000123146	ENSRNOG00000004489	ENSMUSG00000002885	ADGRE5	15.55	21.00	0.00	0.33	9.36
ENSG00000124493	ENSRNOG00000000487	ENSMUSG00000063239	GRM4	0.08	0.00	0.00	8.07	0.00
ENSG00000125384	ENSRNOG00000034156	ENSMUSG00000037759	PTGER2	5.50	0.00	0.00	0.00	0.11
ENSG00000125734	ENSRNOG00000046128	ENSMUSG00000005823	GPR108	22.19	17.60	0.00	NA#	24.94
ENSG00000126010	ENSRNOG00000004124	ENSMUSG00000031364	GRPR	0.35	0.00	0.00	4.26	0.00
ENSG00000128271	ENSRNOG00000001302	ENSMUSG00000020178	ADORA2A	1.88	1.12	0.00	0.00	0.00

ENSG00000128602	ENSRNOG00000008332	ENSMUSG00000001761	SMO	24.06	2.94	0.00	28.48	14.89
ENSG00000130810	ENSRNOG00000020608	ENSMUSG00000004100	PPAN	67.16	64.63	0.00	17.16	147.78
ENSG00000132911	ENSRNOG00000014081	ENSMUSG00000037393	NMUR2	1.75	0.00	0.00	0.00	0.00
ENSG00000133019	ENSRNOG00000014639	ENSMUSG00000046159	CHRM3	2.23	0.03	3.72	0.15	0.00
ENSG00000133105	ENSRNOG00000000897	ENSMUSG00000053368	RXFP2	0.00	0.00	0.00	5.93	0.00
ENSG00000134830	ENSRNOG00000049028	ENSMUSG00000074361	C5AR2	0.00	0.00	0.00	NA#	2.46
ENSG00000135312	ENSRNOG00000013042	ENSMUSG00000049511	HTR1B	0.27	0.00	0.00	5.08	0.36
ENSG00000136928	ENSRNOG00000008431	ENSMUSG00000039809	GABBR2	1.12	0.00	0.00	0.13	0.00
ENSG00000137819	ENSRNOG00000014164	ENSMUSG00000032278	PAQR5	0.88	1.76	9.16	0.00	0.06
ENSG00000138271	ENSRNOG00000013894	ENSMUSG00000051431	GPR87	1.80	55.21	0.00	0.00	0.00
ENSG00000139292	ENSRNOG00000004221	ENSMUSG00000020140	LGR5	8.90	0.00	0.00	0.00	0.00
ENSG00000139572	ENSRNOG00000036834	ENSMUSG00000063234	GPR84	0.00	0.17	0.25	0.00	36.23
ENSG00000139679	ENSRNOG00000015577	ENSMUSG00000033446	LPAR6	1.06	0.00	0.15	6.77	11.84
ENSG00000140030	ENSRNOG00000003806	ENSMUSG00000021886	GPR65	0.00	0.00	0.00	0.00	17.51
ENSG00000143126	ENSRNOG00000020058	ENSMUSG00000068740	CELSR2	9.28	16.79	14.97	2.78	0.00
ENSG00000143147	ENSRNOG00000003073	ENSMUSG00000040836	GPR161	6.79	3.86	6.46	5.92	0.20

ENSG00000144230	ENSRNOG00000025745	ENSMUSG00000052229	GPR17	0.17	0.00	0.00	1.64	0.00	
ENSG00000144407	ENSRNOG00000015259	ENSMUSG00000025946	PTH2R	0.00	3.59	0.00	0.00	0.00	
ENSG00000144476	ENSRNOG00000019622	ENSMUSG00000044337	ACKR3	0.00	41.54	0.00	168.11	0.00	
ENSG00000148358	ENSRNOG00000023589	ENSMUSG00000000194	GPR107	22.09	19.96	0.00	24.79	17.75	
ENSG00000148680	ENSRNOG00000018827	ENSMUSG00000024798	HTR7	0.64	0.85	1.04	0.13	0.00	
ENSG00000148926	ENSRNOG00000027030	ENSMUSG00000030790	ADM	11.00	11.79	71.50	2.85	0.16	
ENSG00000150471	ENSRNOG00000030149	ENSMUSG00000037605	ADGRL3	5.82	0.16	0.00	0.03	0.00	
ENSG00000151025	ENSRNOG00000024832	ENSMUSG00000045967	GPR158	0.03	2.29	2.65	0.00	0.00	
ENSG00000152749	ENSRNOG00000009766	ENSMUSG00000022131	GPR180	9.68	15.28	22.26	9.80	8.45	
ENSG00000152822	ENSRNOG00000014290	ENSMUSG00000019828	GRM1	0.00	0.00	0.00	1.55	0.00	
ENSG00000152990	ENSRNOG00000004279	ENSMUSG00000029090	ADGRA3	19.59	15.04	0.00	13.37	0.00	
ENSG00000153294	ENSRNOG00000012535	ENSMUSG00000023918	ADGRF4	2.09	2.22	0.00	0.00	0.00	
ENSG00000155760	ENSRNOG00000016119	ENSMUSG00000041075	FZD7	5.86	7.15	0.00	47.44	2.27	
ENSG00000156097	ENSRNOG00000019738	ENSMUSG00000046793	GPR61	0.44	2.11	0.14	0.00	0.15	
ENSG00000157240	ENSRNOG00000016242	ENSMUSG00000044674	FZD1	5.43	3.76	0.00	24.79	0.00	
ENSG00000158292	ENSRNOG00000010718	ENSMUSG00000042804	GPR153	3.11	2.15	5.35	0.39	0.00	

ENSG00000159346	ENSRNOG00000004143	ENSMUSG00000026457	ADIPOR1	66.14	50.78	301.05	81.69	28.69
ENSG00000160013	ENSRNOG00000016756	ENSMUSG00000043017	PTGIR	0.00	0.00	0.00	0.35	21.36
ENSG00000160683	ENSRNOG00000012430	ENSMUSG00000047880	CXCR5	0.00	0.00	0.00	0.00	1.60
ENSG00000160781	ENSRNOG00000026059	ENSMUSG00000041423	PAQR6	1.20	3.90	2.70	12.88	0.00
ENSG00000160951	ENSRNOG00000004094	ENSMUSG00000019464	PTGER1	0.15	0.00	0.00	8.69	14.73
ENSG00000162073	ENSRNOG00000003721	ENSMUSG00000023909	PAQR4	11.45	34.15	5.49	0.47	8.79
ENSG00000163251	ENSRNOG00000014678	ENSMUSG00000045005	FZD5	5.29	4.06	0.00	2.84	0.62
ENSG00000163291	ENSRNOG00000002035	ENSMUSG00000055725	PAQR3	18.36	10.81	0.00	3.08	5.76
ENSG00000163328	ENSRNOG00000018485	ENSMUSG00000041762	GPR155	1.94	0.54	0.00	0.08	3.33
ENSG00000163331	ENSRNOG00000005743	ENSMUSG00000026989	DAPL1	0.00	1.76	0.00	0.00	0.00
ENSG00000163485	ENSRNOG00000003442	ENSMUSG00000042429	ADORA1	1.27	0.00	0.90	0.00	0.00
ENSG00000163870	ENSRNOG00000016665	ENSMUSG00000002871	TPRA1	20.12	49.79	0.00	19.32	26.62
ENSG00000163933	ENSRNOG00000021805	ENSMUSG00000052395	RFT1	26.11	77.92	0.00	6.99	9.60
ENSG00000164199	ENSRNOG00000016306	ENSMUSG00000069170	ADGRV1	7.60	0.81	0.00	0.13	0.83
ENSG00000164251	ENSRNOG00000018003	ENSMUSG00000021678	F2RL1	53.08	17.74	0.00	1.11	0.00
ENSG00000164849	ENSRNOG00000001288	ENSMUSG00000044197	GPR146	6.33	0.76	0.00	1.69	3.30

ENSG00000164930	ENSRNOG00000004660	ENSMUSG00000022297	FZD6	21.51	57.19	0.00	1.03	0.10
ENSG00000166073	ENSRNOG00000005971	ENSMUSG00000040133	GPR176	5.34	4.33	0.84	0.10	0.05
ENSG00000166148	ENSRNOG00000004400	ENSMUSG00000020123	AVPR1A	0.18	0.00	0.17	65.55	0.00
ENSG00000167191	ENSRNOG00000016013	ENSMUSG00000008734	GPRC5B	4.23	0.05	0.99	0.36	0.00
ENSG00000168329	ENSRNOG00000018509	ENSMUSG00000052336	CX3CR1	0.06	0.00	0.00	0.00	183.97
ENSG00000168398	ENSRNOG00000004454	ENSMUSG00000021070	BDKRB2	0.46	2.82	1.87	4.12	0.00
ENSG00000169252	ENSRNOG00000019217	ENSMUSG00000045730	ADRB2	1.18	14.56	0.00	2.56	12.30
ENSG00000169403	ENSRNOG00000013231	ENSMUSG00000056529	PTAFR	0.69	5.78	0.73	1.60	6.37
ENSG00000169508	ENSRNOG00000025094	ENSMUSG00000051212	GPR183	0.00	0.14	1.74	0.13	39.58
ENSG00000169860	ENSRNOG00000014232	ENSMUSG00000027765	P2RY1	1.00	1.66	0.00	0.00	0.00
ENSG00000169962	ENSRNOG00000019589	ENSMUSG00000029072	TAS1R3	0.11	0.34	1.43	0.14	0.17
ENSG00000170214	ENSRNOG00000060087	ENSMUSG00000050541	ADRA1B	1.30	0.00	0.00	NA#	0.00
ENSG00000170412	ENSRNOG00000003144	ENSMUSG00000051043	GPRC5C	21.67	4.82	0.00	25.69	0.29
ENSG00000170425	ENSRNOG00000002922	ENSMUSG00000018500	ADORA2B	11.22	17.00	1.49	0.31	3.51
ENSG00000170775	ENSRNOG00000002524	ENSMUSG00000039904	GPR37	4.01	0.47	0.00	0.00	0.00
ENSG00000170837	ENSRNOG00000010880	ENSMUSG00000072875	GPR27	9.16	0.00	0.00	0.00	0.00

ENSG00000170915	ENSRNOG00000012830	ENSMUSG00000025931	PAQR8	0.94	0.79	0.00	5.46	0.00
ENSG00000170989	ENSRNOG00000013683	ENSMUSG00000045092	S1PR1	0.91	0.00	0.36	3.68	13.47
ENSG00000171517	ENSRNOG00000015260	ENSMUSG00000036832	LPAR3	0.58	23.85	0.31	0.00	0.00
ENSG00000171522	ENSRNOG00000013240	ENSMUSG00000039942	PTGER4	2.63	0.77	0.00	0.00	5.45
ENSG00000171631	ENSRNOG00000019270	ENSMUSG00000048779	P2RY6	0.29	0.18	10.09	0.90	46.64
ENSG00000171860	ENSRNOG00000009211	ENSMUSG00000040552	C3AR1	1.54	0.23	0.00	0.00	17.67
ENSG00000173198	ENSRNOG00000037845	ENSMUSG00000052821	CYSLTR1	0.58	0.39	0.00	0.00	2.40
ENSG00000173264	ENSRNOG00000021145	ENSMUSG00000024958	GPR137	30.15	12.88	102.12	10.73	10.17
ENSG00000173567	ENSRNOG00000024766	ENSMUSG00000067642	ADGRF3	0.27	0.00	0.00	0.00	1.56
ENSG00000173698	ENSRNOG00000032472	ENSMUSG00000031298	ADGRG2	2.31	0.00	0.00	0.04	0.00
ENSG00000173890	ENSRNOG00000009487	ENSMUSG00000037661	GPR160	11.04	1.25	0.00	0.17	0.13
ENSG00000174804	ENSRNOG00000016848	ENSMUSG00000049791	FZD4	2.40	1.01	2.71	3.07	0.00
ENSG00000174837	ENSRNOG00000046254	ENSMUSG00000004730	ADGRE1	0.00	0.00	0.00	NA#	36.63
ENSG00000175591	ENSRNOG00000019283	ENSMUSG00000032860	P2RY2	1.26	6.28	7.93	0.00	2.83
ENSG00000177283	ENSRNOG00000038571	ENSMUSG00000036904	FZD8	4.52	0.00	2.20	0.37	0.06
ENSG00000178201	ENSRNOG00000032657	ENSMUSG00000061602	VN1R1	2.59	0.00	0.00	0.00	0.00

	ENSRNOG00000030899	ENSMUSG00000047655	VN1R1	2.59	0.00	0.00	0.00	0.00
		ENSMUSG00000057161	VN1R1	2.59	0.00	0.00	0.00	0.00
		ENSMUSG00000043308	VN1R1	2.59	0.00	0.00	0.00	0.00
		ENSMUSG00000045713	VN1R1	2.59	0.00	0.00	0.00	0.00
		ENSMUSG00000058132	VN1R1	2.59	0.00	0.00	0.00	0.00
		ENSMUSG00000091539	VN1R1	2.59	0.00	0.00	0.00	0.00
		ENSMUSG00000066803	VN1R1	2.59	0.00	0.00	0.00	0.00
		ENSMUSG00000066804	VN1R1	2.59	0.00	0.00	0.00	0.00
ENSG00000178623	ENSRNOG00000024030	ENSMUSG00000026271	GPR35	0.27	0.00	0.00	0.00	4.28
ENSG00000179546	ENSRNOG00000012038	ENSMUSG00000070687	HTR1D	2.16	0.22	0.64	0.00	0.00
ENSG00000179603	ENSRNOG00000021468	ENSMUSG00000024211	GRM8	2.12	0.00	0.00	1.29	0.00
ENSG00000180340	ENSRNOG00000021962	ENSMUSG00000050288	FZD2	9.28	11.00	9.45	51.94	0.11
ENSG00000180616	ENSRNOG00000002793	ENSMUSG00000047904	SSTR2	1.22	1.62	0.00	0.00	0.14
ENSG00000180720	ENSRNOG00000017556	ENSMUSG00000040495	CHRM4	0.14	0.00	0.00	0.00	2.92
ENSG00000180739	ENSRNOG00000020901	ENSMUSG00000045087	S1PR5	2.56	5.11	0.00	0.10	0.00
ENSG00000180758	ENSRNOG00000017528	ENSMUSG00000047875	GPR157	7.38	20.30	7.55	0.64	0.51

ENSG00000180998	ENSRNOG00000026328	ENSMUSG00000049092	GPR137C	4.29	0.00	15.26	0.16	0.00
ENSG00000181104	ENSRNOG00000025411	ENSMUSG00000048376	F2R	20.78	1.76	0.00	63.46	0.00
ENSG00000181619	ENSRNOG00000004499	ENSMUSG00000043398	GPR135	1.28	1.06	0.68	0.00	0.49
ENSG00000181773	ENSRNOG00000009540	ENSMUSG00000049649	GPR3	2.18	5.58	1.03	0.00	0.00
ENSG00000182749	ENSRNOG00000022054	ENSMUSG00000037348	PAQR7	2.89	14.93	30.59	4.41	15.95
ENSG00000182782	ENSRNOG00000026653	ENSMUSG00000045502	HCAR2	0.00	14.76	1.69	0.00	0.55
ENSG00000183150	ENSRNOG00000007126	ENSMUSG00000032641	GPR19	0.57	1.26	6.17	1.67	6.28
ENSG00000183484	ENSRNOG00000013914	ENSMUSG00000021298	GPR132	0.99	0.35	1.55	0.00	5.69
ENSG00000183729	ENSRNOG00000007640	ENSMUSG00000033774	NPBWR1	0.22	1.23	0.00	0.00	0.00
ENSG00000183813	ENSRNOG00000010315	ENSMUSG00000047898	CCR4	1.39	0.00	0.00	0.00	0.07
ENSG00000183840	ENSRNOG00000021586	ENSMUSG00000026343	GPR39	0.21	3.73	0.00	0.00	0.00
ENSG00000184160	ENSRNOG00000009299	ENSMUSG00000045318	ADRA2C	1.89	0.00	0.00	0.00	0.00
ENSG00000184194	ENSRNOG00000003272	ENSMUSG00000056679	GPR173	1.31	0.00	0.00	3.28	0.00
ENSG00000184451	ENSRNOG00000020275	ENSMUSG00000044052	CCR10	0.00	0.00	7.68	0.55	3.15
ENSG00000184574	ENSRNOG00000021401	ENSMUSG00000067714	LPAR5	1.44	1.55	0.92	0.00	10.66
ENSG00000186188	ENSRNOG00000021763	ENSMUSG00000054200	FFAR4	0.05	0.00	0.00	0.00	3.85

ENSG00000186810	ENSRNOG00000003305	ENSMUSG00000050232	CXCR3	0.00	0.00	0.00	0.00	3.77
ENSG00000186912	ENSRNOG00000002953	ENSMUSG00000044359	P2RY4	0.00	0.00	0.00	3.97	0.00
ENSG00000188092	ENSRNOG00000000095	ENSMUSG00000028096	GPR89B	0.63	0.63	1.08	15.02	29.61
ENSG00000188763	ENSRNOG00000001452	ENSMUSG00000049551	FZD9	4.59	0.00	0.00	0.16	0.28
ENSG00000188822	ENSRNOG00000009260	ENSMUSG00000062585	CNR2	0.00	0.00	1.67	0.00	2.48
ENSG00000196341	ENSRNOG00000012079	ENSMUSG00000058515	OR8D1	0.00	0.00	0.00	0.00	4.72
		ENSMUSG00000063221	OR8D1	0.00	0.00	0.00	0.00	0.52
		ENSMUSG00000047667	OR8D1	0.00	0.00	0.00	0.00	0.00
ENSG00000196639	ENSRNOG00000007420	ENSMUSG00000053004	HRH1	0.67	3.00	0.00	0.00	0.00
ENSG00000197081	ENSRNOG00000014997	ENSMUSG00000023830	IGF2R	45.96	51.40	0.00	25.78	21.70
ENSG00000197405	ENSRNOG00000047800	ENSMUSG00000049130	C5AR1	0.17	0.00	0.00	NA#	34.97
ENSG00000198121	ENSRNOG00000013656	ENSMUSG00000038668	LPAR1	5.21	3.79	0.00	0.35	0.00
ENSG00000204681	ENSRNOG00000000774	ENSMUSG00000024462	GABBR1	26.16	9.50	0.00	46.49	0.00
ENSG00000205213	ENSRNOG00000005715	ENSMUSG00000050199	LGR4	19.76	13.14	73.27	42.54	0.61
ENSG00000205336	ENSRNOG00000014963	ENSMUSG00000031785	ADGRG1	0.61	21.51	158.08	10.53	0.06
ENSG00000212127	ENSRNOG00000033896	ENSMUSG00000071147	TAS2R14	3.18	0.60	1.37	0.00	0.00

	ENSRNOG00000032724	ENSMUSG00000030196	TAS2R14	3.18	0.60	1.37	0.00	0.00
	ENSRNOG00000021366	ENSMUSG00000057381	TAS2R14	3.18	0.60	1.37	0.00	0.00
	ENSRNOG00000031836	ENSMUSG00000063762	TAS2R14	3.18	0.60	1.37	0.00	0.00
	ENSRNOG00000021329	ENSMUSG00000062952	TAS2R14	3.18	0.60	1.37	0.00	0.00
	ENSRNOG00000021397	ENSMUSG00000056926	TAS2R14	3.18	0.60	1.37	0.46	0.00
	ENSRNOG00000030752	ENSMUSG00000059410	TAS2R14	3.18	0.60	1.37	0.00	0.00
	ENSRNOG00000031107	ENSMUSG00000062528	TAS2R14	3.18	0.60	1.37	0.00	0.00
		ENSMUSG00000030194	TAS2R14	3.18	0.60	1.37	0.00	0.00
		ENSMUSG00000058349	TAS2R14	3.18	0.60	1.37	0.00	0.00
ENSG00000213694	ENSRNOG00000014524	ENSMUSG00000067586	S1PR3	3.79	0.00	0.00	12.72	0.05
ENSG00000213903	ENSRNOG00000020399	ENSMUSG00000046908	LTB4R	9.81	7.99	6.97	0.00	0.19
ENSG00000213906	ENSRNOG00000020382	ENSMUSG00000040432	LTB4R2	6.78	9.22	7.44	0.00	0.00
ENSG00000221836	ENSRNOG00000005407	ENSMUSG00000043119	OR2A5	0.00	0.00	0.00	5.70	0.00
ENSG00000238243	ENSRNOG00000038413	ENSMUSG00000060030	OR2W3	1.91	0.54	5.52	0.00	0.00
		ENSMUSG00000063549	OR2W3	1.91	0.54	5.52	0.00	0.00
ENSG00000243207	ENSRNOG00000020608	ENSMUSG00000004100	PPAN-P2RY11	2.40	0.00	0.00	17.16	147.78

ENSG00000243729	ENSRNOG00000000759	ENSMUSG00000090675	OR5V1	0.00	0.00	0.00	0.00	1.36
ENSG00000243729	ENSRNOG00000000759	ENSMUSG00000090894	OR5V1	0.00	0.00	0.00	0.00	0.21
ENSG00000250510	ENSRNOG00000016143	ENSMUSG00000038390	GPR162	1.36	0.00	0.00	1.50	1.27
ENSG00000255398	ENSRNOG00000026653	ENSMUSG00000045502	HCAR3	0.00	1.91	0.00	0.00	0.55
ENSG00000175898	ENSRNOG00000020653	ENSMUSG00000043895	S1PR2	3.29	1.04	0.00	12.51	10.35

[#] NA no orthologous annotated

Supplemental Table S3: List of structurally related GPCRs of the P2Y group with their expression in the tested cell

receptor	alias	Ensembl ID (human)	tested in orphan Pannel (DiscoveRx)	H1819	RAW264.7	HEK293	A431	A7r5
GPR171	H963	ENSG00000174946	yes	no	no	no	no	no
P2RY12		ENSG00000169313	no	no	no	no	no	no
P2RY13	GPCR1, GPR94, Gpr86	ENSG00000181631	no	no	no	no	no	no
GPR87		ENSG00000138271	no	no	no	yes	yes	no
P2RY14	GPR105	ENSG00000174944	no	no	no	no	no	no
GPR82		ENSG00000171657	no	no	no	no	no	no
GPR34		ENSG00000171659	no	no	no	no	no	no
P2RY10	LYPSR2	ENSG00000078589	no	no	no	no	no	no
PAFR	PTAFR	ENSG00000169403	no	no	yes	no	yes	yes
GPR35		ENSG00000178623	no	no	yes	no	no	no
GPR92	LPAR5	ENSG00000184574	no	no	yes	yes	yes	no
P2RY5	LPAR6	ENSG00000139679	no	no	yes	yes	no	yes
GPR23	LPA4; P2RY9	ENSG00000147145	yes	no	no	no	no	no
GPR21	CysLTR2	ENSG00000188394	no	no	no	no	no	no
CysLTR1		ENSG00000173198	no	no	yes	no	no	no
P2RY11		ENSG00000244165	no	no	NA#	yes	yes	NA#
P2RY1		ENSG00000169860	no	no	no	yes	yes	no
GPR99	OXGR1	ENSG00000165621	yes	no	no	no	no	no
GPR91	SUCNR1	ENSG00000198829	yes	no	no	no	no	no
P2RY4	NRU; P2P; UNR	ENSG00000186912	no	no	no	no	no	yes
P2RY2		ENSG00000175591	no	yes	yes	yes	yes	no
GPR17		ENSG00000144230	yes	no	no	no	no	yes
P2RY6		ENSG00000171631	no	yes	yes	no	no	no

NA no orthologues annotated

Supplemental Figure S1

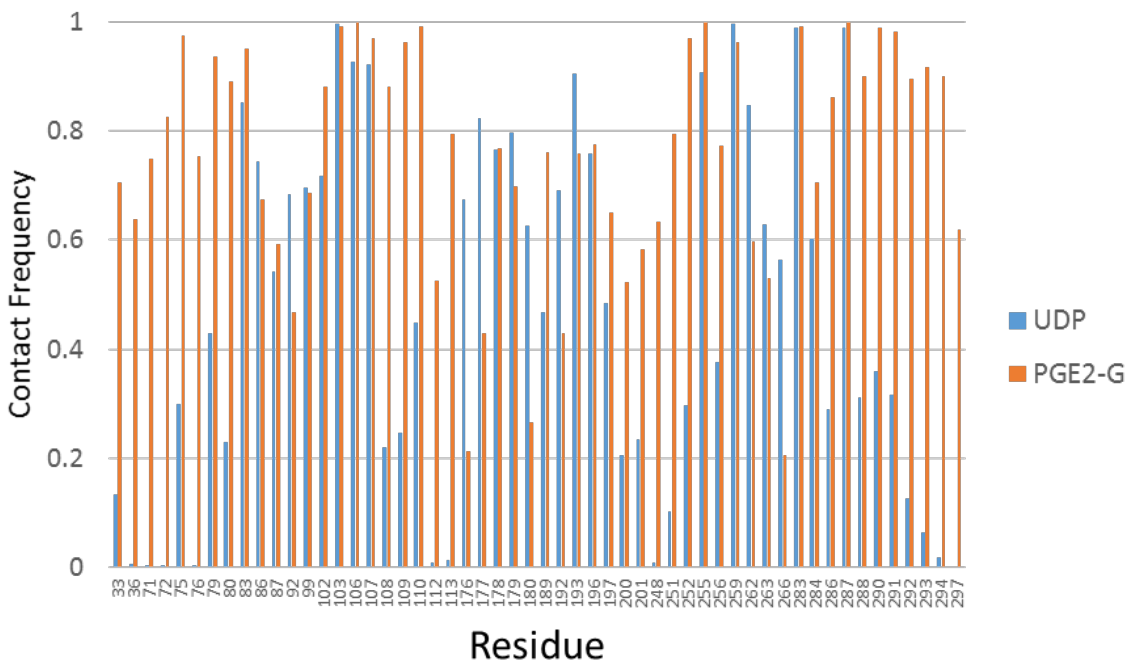

Contact frequencies were assessed across all equally scoring poses in a ligand pose ensemble. Residues contacting a ligand across the majority of the poses in an ensemble were considered potential contact points for the corresponding ligand.
